# Three decades of household food availability according to NOVA - Brazil, 1987–2018

**DOI:** 10.11606/s1518-8787.2022056004570

**Published:** 2022-08-01

**Authors:** Renata Bertazzi Levy, Giovanna Calixto Andrade, Gabriela Lopes da Cruz, Fernanda Rauber, Maria Laura da Costa Louzada, Rafael Moreira Claro, Carlos Augusto Monteiro

**Affiliations:** I Universidade de São Paulo Faculdade de Medicina Departamento de Medicina Preventiva São Paulo SP Brasil Universidade de São Paulo . Faculdade de Medicina . Departamento de Medicina Preventiva . São Paulo , SP , Brasil; II Universidade de São Paulo Núcleo de Pesquisas Epidemiológicas em Nutrição e Saúde São Paulo SP Brasil Universidade de São Paulo . Núcleo de Pesquisas Epidemiológicas em Nutrição e Saúde . São Paulo , SP , Brasil; III Universidade de São Paulo Faculdade de Saúde Pública Programa de Pós-Graduação em Nutrição em Saúde Pública São Paulo SP Brasil Universidade de São Paulo . Faculdade de Saúde Pública . Programa de Pós-Graduação em Nutrição em Saúde Pública . São Paulo , SP , Brasil; IV Universidade de São Paulo Faculdade de Saúde Pública Departamento de Nutrição São Paulo SP Brasil Universidade de São Paulo . Faculdade de Saúde Pública . Departamento de Nutrição . São Paulo , SP , Brasil; V Universidade Federal de Minas Gerais Escola de Enfermagem Departamento de Nutrição Belo Horizonte MG Brasil Universidade Federal de Minas Gerais . Escola de Enfermagem . Departamento de Nutrição . Belo Horizonte , MG , Brasil

**Keywords:** Diet, Food, and Nutrition, Staple Food, Industrialized Foods, Socioeconomic Factors, Food Economics

## Abstract

**OBJECTIVE:**

To evaluate the trend of household food acquisition according to the NOVA classification in Brazil between 1987–1988 and 2017–2018.

**METHODS:**

We used household food acquisition data from five editions of the *Pesquisas de Orçamentos Familiares* (Household Budget Surveys), conducted by the *Instituto Brasileiro de Geografia e Estatística* (Brazilian Institute of Geography and Statistics), in the years 1987–1988, 1995–1996, 2002–2003, 2008–2009, and 2017–2018. All reported foods were categorized according to the NOVA classification. The household availability of food groups and subgroups was expressed through their share (%) in total calories, for all Brazilian families, by household situation (urban or rural), for each of the five geographic regions of the country, by fifths of the household income *per capita* distribution (2002–2003, 2008–2009 and 2017–2018 surveys), and for the 11 main urban regions of the country (1987–1988, 1995–1996, 2002–2003, 2008–2009 and 2017–2018 surveys). Linear regression models were used to assess the trend of increasing or decreasing food purchases.

**RESULTS:**

The diet of the Brazilian population is still composed predominantly of foods *in natura* or minimally processed and processed culinary ingredients. However, our findings point to trends of increasing share of ultra-processed foods in the diet. This increase of 0.4 percentage points per year between 2002 and 2009 slowed down to 0.2 percentage points between 2008 and 2018. The consumption of ultra-processed food was higher among households with higher income, in the South and Southeast regions, in urban areas, and in metropolitan regions.

**CONCLUSION:**

Our results indicate an increase in the share of ultra-processed foods in the diet of Brazilians. This is a worrisome scenario, since the consumption of such foods is associated with the development of diseases and the loss of nutritional quality of the diet.

## INTRODUCTION

Several international organizations such as the World Health Organization (WHO) and the Food and Agriculture Organization (FAO) advocate that healthy and sustainable eating patterns are those based on a wide variety of foods *in natura* or minimally processed and restricted in highly processed foods ^1^ . Although there is more than one way to classify food items according to the processing they have undergone, the NOVA system is by far the most widely used ^2^ , according to which foods are classified according to the extent and purpose of their industrial processing into four major groups: foods *in natura* or minimally processed, processed culinary ingredients, processed foods, and ultra-processed foods ^3^ . Based on this classification, the *Guia Alimentar para a População Brasileira* (Food Guide for the Brazilian Population), published in 2014, recommends: that the diet be based on a wide variety of foods *in natura* or minimally processed; that processed culinary ingredients be used in small quantities to transform foods from the first group into culinary preparations; that processed foods, also in small quantities, be used as part of culinary preparations or accompaniments; and that the consumption of ultra-processed foods be avoided ^4^ .

Numerous evidence, including systematic reviews of cohort studies and with meta-analysis, shows that higher consumption of ultra-processed foods is associated with the risk of non-communicable chronic diseases, such as obesity, hypertension, diabetes, dyslipidemias, cardiovascular diseases, depression, cancers - such as breast cancer -, gastrointestinal disorders, as well as early mortality from all causes ^2 , 5^ .

In Brazil, the evolution of household food availability classified according to the NOVA system has been documented based on the *Pesquisas de Orçamentos Familiares* (POF - Household Budget Surveys) carried out by the *Instituto Brasileiro de Geografia e Estatística* (IBGE - Brazilian Institute of Geography and Statistics) since 1987–1988, in metropolitan areas, and since 2002–2003, in the country as a whole. The share of ultra-processed products in household food purchases increased both in metropolitan areas between 1987–1988 and 2002–2003, and for the country as a whole between 2002–2003 and 2008–2009, with important variations according to location and household income level ^11^ .

A new POF by IBGE in 2017–2018 allows updating the evolution trend of the household acquisition of food in Brazil, classified according to the extent and purpose of its processing, which is presented below in this article.

## METHODS

This study used household food acquisition data from five editions of POF, conducted by IBGE, in the periods March 1987 to February 1988, October 1995 to September 1996, June 2002 to July 2003, May 2008 to May 2009, and July 2017 to July 2018.

In the research first two editions, representative samples of Brazilian households located in the main urban regions of Brazil (metropolitan region of Belém in the North region, Fortaleza, Recife and Salvador in the Northeast region; Belo Horizonte, Rio de Janeiro and São Paulo in the Southeast region; Curitiba and Porto Alegre in the South region; and the Distrito Federal and the municipality of Goiânia, in the Center-West region). In the three latest editions of the survey, the sample was expanded to represent, in addition to these domains, the complete set of households in the country. The surveys used a complex sampling plan, by clusters in two stages, involving the random selection of census sectors in the first stage and households in the second. The census sectors come from the IBGE’s master sample, grouped in strata of households with high geographical and socioeconomic homogeneity. For the construction of the strata, the following were considered: the geographic location of the sector; the situation of the household (urban or rural for samples with national representation); and, within each geographic locus, the spectrum of socioeconomic variation through the income of the individual responsible for the household.

The estimates obtained in the surveys with national samples represent the following domains: the country, the five large regions (North, Northeast, Southeast, South, and Midwest), the situation (urban or rural), the 26 federal units and the Distrito Federal, the nine metropolitan regions, and the 26 state capitals. A detailed description of the sampling process for the five surveys is available in IBGE’s publications ^a^ .

For the present study, the household clusters generated in the sampling plan (strata) were used as the unit of analysis. For the nationally representative analyses, in 2002–2003 the 48,747 households resulted in 443 strata with an average of 109.4 households per stratum (ranging from nine to 801); in 2008–2009 the 55,970 households generated 550 strata with an average of 101.7 households per stratum (ranging from eight to 796) and in 2017–2018 the 57,920 households resulted in 575 strata with an average of 86.5 households per stratum (ranging from 16 to 524).

For the trend analyses of the metropolitan regions, we used information from the IBGE System of Automatic Recovery (SIDRA) ^b^ on food and beverage purchases in each of the nine metropolitan regions, the municipality of Goiânia and the Distrito Federal. In SIDRA, information is available on household clusters corresponding to 10 family income classes, totaling 110 strata per survey.

The information used in this study refers to food purchases for home consumption made during seven consecutive days, recorded by the residents of the household or by an IBGE interviewer in a collective expenditure booklet (in home measurements or in the acquisition unit itself) and converted into kilograms or liters by IBGE. We failed to cover data on food consumed outside the house with a satisfactory level of detail, so we left it out of this study.

Data collection for each survey was spread over the four quarters of the year, incorporating the seasonal fluctuation to which expenses are subject. We defined the acquisition quantities for the 1987–1988 survey indirectly by the relationship between expenses and reported items, due to the absence of data collected in this period.

Correction factors were applied to exclude the inedible fraction of the food ^c^ . Then, we converted the edible amount of food into calories using the Brazilian Table of Food Composition ^d^ .

All reported foods were categorized according to the four groups of the NOVA classification: 1) Foods *in natura* or minimally processed; 2) Processed culinary ingredients; 3) Processed foods; and 4) Ultra-processed foods ^3^ .

Household food availability of the groups and subgroups was expressed by their share (%) in the calories available for consumption. The share of food groups and subgroups was estimated for all Brazilian families, by household situation (urban or rural) for each of the five geographic regions of the country and according to fifths of the distribution of household income *per capita* (2002–2003, 2008–2009 and 2017–2018 surveys), and for those residing in the 11 main urban regions of the country (1987–1988, 1995–1996, 2002–2003, 2008–2009 and 2017–2018 surveys).

We used linear regression models to evaluate the trend and the difference between the years in the share of foods in the diet of Brazilians during the period studied, with the first year of the survey as the explanatory variable and the groups and subgroups of foods as the outcome. We considered p < 0.05 for statistical significance in all analyses.

Weighting factors were used, considering the sample structure and expansion factors, allowing extrapolation of the results to the Brazilian population. All analyses were performed with the statistical package of the Stata software (StataCorp, version 16).

## RESULTS

Table 1 describes the relative share of food groups and subgroups, according to the NOVA classification, in household food availability in 2002–2003, 2008–2009 and 2017–2018 in Brazil. In 2017–2018 48.7% of the calories available for consumption in Brazilian households were from foods *in natura* or minimally processed, 21.6% from processed culinary ingredients, 10.4% from processed foods, and 19.4% from ultra-processed foods.

**Table 1:** Relative share of NOVA classification groups and subgroups in total calories determined by household food purchases. Brazil – periods 2002–2003, 2008–2009 e 2017–2018.

Food groups and subgroups	Relative share, per survey year (%)		
	2002–2003	2008–2009	2017–2018
**Foods in natura or minimally processed**	**51.0**	**48.9** ^e^	**48.7** ^f^
Rice	16.5	15.4	15.1 ^f^
Milk	5.4	4.8 ^e^	4.7 ^f^
Poultry	3.1	3.6 ^e^	4.1 ^e,f^
Beans	5.5	4.6 ^e^	4.1 ^e,f^
Beef	3.3	4.3 ^e^	4.6 ^e,f^
Fruit	2.1	2.4 ^e^	2.8 ^e,f^
Pasta	2.4	2.4	2.4
Cornmeal	1.6	1.4	1.1 ^e,f^
Cassava flour	3.6	2.7	1.6 ^e,f^
Wheat flour	2.6	1.9 ^e^	1.7 ^f^
Roots and tubers	1.1	1.1	1.2 ^e,f^
Eggs	0.3	0.7 ^e^	0.9 ^e,f^
Vegetables	0.7	0.8 ^e^	0.9 ^e,f^
Pork	0.7	0.6	1.0 ^e,f^
Fish	0.5	0.5	0.4
Corn, oats and other cereals	0.7	0.9	1.0 ^e,f^
Viscera	0.3	0.2	0.2
Other ^a^	0.5	0.6 ^e^	0.7 ^f^
**Processed culinary ingredients**	**25.5**	**23.4** ^e^	**21.6** ^e,f^
Vegetable oil	11.7	11.0	10.7 ^f^
Sugar	12.5	11.3 ^e^	9.4 ^e,f^
Animal fat	0.8	0.5 ^e^	0.7 ^e^
Starches	0.4	0.5	0.7 ^e,f^
Other ^b^	0.1	0.1	0.1 ^e,f^
**Processed foods**	**9.2**	**10.4** ^e^	**10.4** ^f^
Bread	6.6	7.4 ^e^	7.0
Cheese	0.9	1.1 ^e^	1.4 ^e,f^
Salted/dried/smoked meats	0.7	0.7	0.7
Fermented alcoholic beverages	0.4	0.6 ^e^	0.7 ^e,f^
Other ^c^	0.5	0.5	0.6 ^f^
**Ultra-processed foods**	**14.3**	**17.3** ^e^	**19.4** ^e,f^
Cold cuts and sausages	2.0	2.4 ^e^	2.7 ^e,f^
Sweet cookies	1.9	2.1 ^e^	2.2 ^f^
Salted biscuits	1.4	1.6 ^e^	1.9 ^e,f^
Margarine	1.8	1.9 ^e^	1.8
Cakes and sweet pies	0.7	1.1 ^e^	1.4 ^e,f^
Breads	0.9	1.1 ^e^	1.4 ^e,f^
Sweets in general	0.5	0.7 ^e^	0.9 ^e,f^
Carbonated sweetened beverages	1.6	1.6	1.2 ^e,f^
Chocolate	0.8	1.0 ^e^	1.2 ^f^
Pizza, lasagna or pastry	0.4	0.6 ^e^	0.9 ^e,f^
Ready meals	0.4	0.7 ^e^	0.9 ^e,f^
Non-carbonated sweetened beverages	0.4	0.5	0.6 ^e,f^
Dairy beverages	0.4	0.5 ^e^	0.5 ^e,f^
Ice cream	0.2	0.3 ^e^	0.4 ^e,f^
Ready-made sauces	0.4	0.5 ^e^	0.7 ^e,f^
Distilled alcoholic beverages	0.1	0.1	0.2 ^f^
Other ^d^	0.4	0.5	0.5

^a^ Other foods *in natura* or minimally processed, including teas and coffees, seafood, meat from other animals, nuts and seeds, and dried or dehydrated fruit and vegetables.

^b^ Other culinary ingredients including salt and other sugars.

^c^ Other processed foods, including dried and/or salted fish and seafood, canned cereals, legumes and vegetables, salted nuts, and canned diet/light meats.

^d^ Other ultra-processed foods, including reconstituted meats, ready-made tablets and seasonings, non-fat salt-based condiments, ultra-processed cheeses, and breakfast cereals.

^e^ p < 0.05 in the comparison with the previous period.

^f^ p < 0.05 for linear trend between 2002–2003 and 2017–2018.

Among foods *in natura* or minimally processed, the groups with the highest caloric contribution were: rice (15.1% of total calories), milk (4.7%), beef (4.6%), beans (4.1%), and poultry (4.1%). Still relevant in the Brazilian diet were fruit (2.8%), pasta (2.4%), wheat flour (1.7%), cassava flour (1.6%), and roots and tubers (1.2%). Among processed culinary ingredients, the subgroups with the highest calorie contribution were vegetable oil (10.7%) and sugar (9.4%). Among processed foods, the subgroups with the highest calorie contribution were bread (7.0%) and cheese (1.4%). Finally, among ultra-processed foods, cold cuts and sausages (2.7%), sweet cookies (2.2%), salted biscuits (1.9%), margarine (1.8%), cakes and sweet pies (1.4%), bread (1.4%), carbonated sweetened beverages (1.2%), and chocolate (1.2%) stand out.

Between 2002–2003 and 2017–2018 we observed a decrease in the percentage calorie share of foods *in natura* or minimally processed (on average -0.15 percentage points per year = pp/year) and processed culinary ingredients (-0.24 pp/year), in parallel with the increase in the share of processed (+0.07 pp/year) and ultra-processed (+0.31 pp/year) foods. The decline in the relative availability of foods *in natura* or minimally processed was more intense in the first period (2002–2003 a 2008–2009) than in the second period (2008–2009 a 2017–2018): 0.30 pp/year and 0.03 pp/year, respectively. A similar evolution was observed for the relative availability of processed culinary ingredients: a decline of 0.3 pp/year in the first period and 0.2 pp/year in the second period. On the other hand, the intensity of the increase in the relative availability of processed and ultra-processed foods decreased from the first to the second period: from 0.2 pp/year to stability for processed foods and from 0.4 pp/year to 0.2 pp/year for ultra- processed foods.

In the group of foods *in natura* or minimally processed, rice (-1.4%), milk (-0.7%), beans (-1.4%), cornmeal (-0.5%), cassava flour (-2.0%) and wheat flour (0.9%) followed the group’s trend, decreasing their share in household availability, while poultry (1.0%), beef (1.3%), fruits (0.7%), roots and tubers vegetables (0.1%), eggs (0.6%), vegetables (0.2%), pork (0.3%), and corn, oats, and other cereals (0.3%) all increased their availability in the period. Among the processed culinary ingredients, there was a drop in the share of vegetable oil (-1.0%) and sugar (-3.1%), as opposed to an increase in starch (+0.3%). The share of cheeses (+0.5%), fermented alcoholic beverages (+0.3%) and other processed foods (+0.1%) increased between 2002–2003 and 2017–2018. In the group of ultra-processed foods, we found an increase in the share of all subgroups, except for margarine, which remained stable, and carbonated sweetened beverages (-0.4%), whose share decreased.

Table 2 describes the availability of food groups and subgroups according to fifths of household income *per capita* between 2002–2003 and 2017–2018. In 2017–2018 the share in total calories of foods *in natura* or minimally processed and processed culinary ingredients decreased with increasing income: in the case of foods *in natura* or minimally processed, from 57.5% in the first fifth to 42.9% in the last; and processed culinary ingredients, from 21.8% in the first fifth to 20.0% in the last. Processed and ultra-processed foods increased their share in total calories along with income. This increase is moderate for processed foods, from 8.8% in the first fifth to 11.8% in the last fifth, and quite intense for ultra-processed foods, from 11.9% to 25.4%. However, the trend of variation in the subgroups with income was heterogeneous. Among foods *in natura* or minimally processed rice, beans and cornmeal decreased their share with the increase in income, while fruits, roots and tubers, and vegetables had their share increased. In the subgroups of processed culinary ingredients, with increasing income, there was a reduction in the caloric share of sugar and an increase in the share of animal fat. Among processed foods, rising incomes led to an increase in the calorie share of cheese, fermented alcoholic beverages, and other processed foods and a reduction in the share of salted/dried/smoked meats. Except for salted biscuits, all other ultra-processed foods increased their share of total calories as income increased.

**Table 2:** Relative share of foods and food groups according to NOVA in the total calories determined by household food acquisition, by fifths of total income and monthly household wealth variation and year of survey - Brazil - periods 2002–2003, 2008–2009 and 2017–2018.

Food groups and subgroups	Relative share, by fifths of total income and monthly family wealth variation (%)														
	1st Fifth			2nd Fifth			3rd Fifth			4th Fifth			5th Fifth		
	2002/03	2008/09	2017/18	2002/03	2008/09	2017/18	2002/03	2008/09	2017/18	2002/03	2008/09	2017/18	2002/03	2008/09	2017/18
**Foods in natura or minimally processed**	**60.0**	**57.6**	**57.5**	**54.4**	**51.3** ^e^	**50.6** ^f^	**50.2**	**47.2** ^e^	**47.3** ^f^	**47.2**	**45.7**	**44.8** ^f^	**43.0**	**42.5**	**42.9**
Rice	17.6	18.2	19.0 ^e^	18.9	16.5 ^e^	16.5 ^f^	18.2	15.8	15.7	15.6	14.6	13.0 ^e,f^	12.1	11.9	11.3
Milk	3.8	3.5	4.1 ^e^	5.0	4.3 ^e^	4.4 ^f^	5.1	5.7	5.2	5.9	5.3 ^e^	5.1 ^f^	7.1	5.2 ^e^	5.0 ^f^
Poultry	2.7	4.0 ^e^	5.7 ^e,f^	3.3	3.8 ^e^	4.3 ^e,f^	3.0	3.6	3.4	3.6	3.2	3.6	2.9	3.2	3.4
Beans	8.5	6.5 ^e^	5.2 ^e,f^	5.8	5.4	4.8 ^e,f^	5.3	4.0 ^e^	3.8 ^f^	4.4	3.6 ^e^	3.3 ^f^	3.5	3.2	3.5
Beef	2.8	4.0 ^e^	4.9 ^e,f^	3.3	4.4 ^e^	4.3 ^f^	3.2	4.6 ^e^	4.6 ^f^	4.0	4.4	4.8 ^f^	3.4	4.0 ^e^	4.2 ^f^
Fruit	1.4	1.8 ^e^	2.3 ^e,f^	1.6	2.1 ^e^	2.5 ^e,f^	1.6	2.2 ^e^	2.8 ^e,f^	2.1	2.4 ^e^	3.0 ^e,f^	3.5	3.5	3.6
Pasta	2.4	2.5	2.5	2.5	2.5	2.6	2.4	2.2	2.3	2.4	2.4	2.2	2.5	2.5	2.5
Cornmeal	3.6	3.1	2.0 ^e,f^	1.8	1.7	1.2 ^e,f^	1.4	0.9 ^e^	0.8 ^f^	0.7	0.7	0.6	0.5	0.6	0.6
Cassava flour	10.5	7.2 ^e^	4.4 ^e,f^	4.0	3.5	2.0 ^e,f^	1.5	1.0	0.6 ^f^	1.0	0.7	0.5 ^f^	0.7	0.8	0.5 ^e,f^
Wheat flour	1.1	0.8	0.8	2.8	1.7 ^e^	1.6 ^f^	3.9	2.2 ^e^	2.0 ^f^	3.2	3.2	2.2 ^e^	2.1	1.9	1.8
Roots and tubers	0.9	0.8	0.9	1.2	1.0	1.2	1.0	1.1	1.4 ^e,f^	1.3	1.3	1.4	1.3	1.2	1.4
Eggs	0.3	0.6 ^e^	0.9 ^e,f^	0.4	0.7 ^e^	1.0 ^e,f^	0.2	0.7 ^e^	0.8 ^e,f^	0.3	0.7 ^e^	0.9 ^e,f^	0.3	0.7 ^e^	1.0 ^e,f^
Vegetables	0.6	0.6	0.7 ^e,f^	0.6	0.8 ^e^	0.9 ^e,f^	0.6	0.8 ^e^	1.0 ^e,f^	0.9	0.8	1.0 ^e,f^	1.0	1.0	1.1
Pork	0.5	0.5	0.7 ^e,f^	1.0	0.6 ^e^	0.8 ^f^	1.0	0.8	1.3 ^e,f^	0.8	0.8	1.1 ^e,f^	0.5	0.6	0.8 ^e,f^
Fish	1.0	1.0	1.0	0.7	0.5	0.4 ^f^	0.2	0.3	0.2	0.3	0.2	0.2	0.3	0.4	0.3
Corn, oats and other cereals	1.3	1.6	1.4	0.7	0.9	1.2 ^f^	1.0	0.6 ^e^	0.6	0.4	0.5	1.0 ^e,f^	0.4	0.7 ^e^	0.9 ^f^
Viscera	0.3	0.3	0.3	0.3	0.3	0.3	0.2	0.2	0.2	0.3	0.2	0.3	0.2	0.2	0.2
Other	0.6	0.7	0.6	0.5	0.6	0.5	0.3	0.4	0.7 ^e,f^	0.4	0.6 ^e^	0.6 ^f^	0.7	0.8	0.8
**Processed culinary ingredients**	**25.1**	**24.8**	**21.8** ^e,f^	**27.2**	**24.4** ^e^	**22.2** ^e,f^	**28.1**	**23.6** ^e^	**22.9** ^f^	**24.7**	**24.2**	**21.0** ^e,f^	**22.2**	**20.2**	**20.0**
Vegetable oil	9.7	9.7	9.3	12.0	10.8	10.4	12.5	11.4	11.9	12.7	12.5	11.2 ^e,f^	11.5	10.8	10.9
Sugar	14.0	13.6	11.0 ^e,f^	13.6	12.6	10.2 ^e,f^	14.1	11.2 ^e^	9.8 ^e,f^	11.0	10.9	8.7 ^e,f^	9.7	8.4 ^e^	7.3 ^e,f^
Animal fat	0.4	0.4	0.4	1.0	0.4 ^e^	0.7 ^e^	1.1	0.7	0.6 ^f^	0.7	0.5	0.7	0.7	0.6	1.1 ^e,f^
Starches	0.7	1.0	1.0 ^f^	0.6	0.5	0.8 ^e,f^	0.3	0.3	0.5 ^e,f^	0.2	0.3 ^e^	0.4 ^f^	0.3	0.3	0.5 ^e,f^
Other ^b^	0.2	0.2	0.1 ^e,f^	0.1	0.1	0.1	0.1	0.1	0.1	0.1	0.1	0.1	0.1	0.1	0.1
**Processed foods**	**6.4**	**7.4**	**8.8** ^f^	**7.9**	**10.8** ^e^	**10.9** ^f^	**8.6**	**11.4** ^e^	**10.0**	**11.0**	**9.7**	**10.6**	**12.2**	**12.4**	**11.8**
Breads	4.5	5.6	6.9 ^f^	5.9	8.2 ^e^	8.2 ^f^	6.4	8.6 ^e^	6.5 ^e^	8.1	6.6	6.4	8.1	7.8	6.8 ^f^
Cheese	0.3	0.4 ^e^	0.6 ^e,f^	0.6	0.8 ^e^	1.1 ^e,f^	0.7	1.1 ^e^	1.4 ^e,f^	1.1	1.3	1.8 ^e,f^	2.0	2.1	2.3
Salted/dried/smoked meats	1.1	0.8 ^e^	0.8	0.7	1.0 ^e^	0.8	0.7	0.7	0.7	0.6	0.5	0.7	0.5	0.6	0.7
Fermented alcoholic beverages	0.1	0.2	0.2	0.3	0.4	0.3	0.4	0.5 ^e^	0.8 ^e,f^	0.5	0.7 ^e^	1.0 ^e,f^	0.9	1.1	1.1
Other ^c^	0.3	0.4 ^e^	0.4	0.4	0.4	0.5 ^f^	0.4	0.5	0.5	0.6	0.6	0.7	0.7	0.8	0.8
**Ultra-processed foods**	**8.6**	**10.2** ^e^	**11.9** ^e,f^	**10.5**	**13.6** ^e^	**16.3** ^e,f^	**13.0**	**17.8** ^e^	**19.9** ^e,f^	**17.2**	**20.4** ^e^	**23.6** ^e,f^	**22.6**	**24.9** ^e^	**25.4** ^f^
Cold cuts and sausages	0.9	1.2 ^e^	1.9 ^e,f^	1.5	2.0 ^e^	2.3 ^e,f^	2.1	3.0 ^e^	2.9 ^f^	3.0	3.0	3.5 ^e,f^	2.5	3.1 ^e^	3.2 ^f^
Sweet cookies	1.6	1.6	1.7	1.5	1.9 ^e^	2.2 ^f^	1.9	2.2	2.2	2.0	2.1	2.4 ^e,f^	2.5	2.6	2.4
Salted biscuits	2.1	2.1	2.3	1.3	1.7 ^e^	2.0 ^f^	1.2	1.3	1.6 ^e,f^	1.1	1.4	1.7 ^e,f^	1.3	1.5	1.9 ^e,f^
Margarine	1.3	1.7 ^e^	1.4 ^e^	1.7	1.8	2.1 ^e^	1.6	2.0 ^e^	1.6 ^e^	1.9	2.0	1.9	2.2	2.3	2.0
Cakes and sweet pies	0.4	0.5	0.8 ^e,f^	0.5	0.8 ^e^	1.1 ^e,f^	0.6	1.0 ^e^	1.6 ^e,f^	0.9	1.5 ^e^	1.6 ^f^	1.2	1.8 ^e^	2.0 ^e,f^
Breads	0.2	0.3	0.5 ^e,f^	0.5	0.7	0.9 ^e,f^	0.7	1.0 ^e^	1.5 ^e,f^	1.0	1.5 ^e^	1.9 ^e,f^	2.0	2.2	2.2
Sweets in general	0.1	0.2 ^e^	0.3 ^e,f^	0.4	0.5	0.7 ^e,f^	0.5	0.8 ^e^	1.0 ^e,f^	0.7	1.0 ^e^	1.2 ^e,f^	1.0	1.3 ^e^	1.4 ^f^
Carbonated sweetened beverages	0.6	0.7	0.6 ^e^	1.1	1.3	0.9 ^e,f^	1.5	1.9 ^e^	1.4 ^e,f^	2.2	2.2	1.6 ^e,f^	2.4	2.1 ^e^	1.5 ^e,f^
Chocolate	0.2	0.3 ^e^	0.4 ^e,f^	0.5	0.6 ^e^	0.8 ^e,f^	0.7	1.0 ^e^	1.3 ^e,f^	1.2	1.4	1.7 ^e,f^	1.6	1.9	1.7
Pizza, lasagna or pastry	0.1	0.2 ^e^	0.3 ^e,f^	0.2	0.4 ^e^	0.5 ^e,f^	0.3	0.6 ^e^	0.9 ^e,f^	0.5	0.8 ^e^	1.2 ^e,f^	1.1	1.0	1.3 ^e,f^
Ready meals	0.1	0.2 ^e^	0.4 ^e,f^	0.2	0.5 ^e^	0.7 ^e,f^	0.4	0.7 ^e^	1.0 ^e,f^	0.5	0.9 ^e^	1.2 ^e,f^	0.9	1.2 ^e^	1.5 ^f^
Non-carbonated sweetened beverages	0.1	0.2 ^e^	0.2 ^e,f^	0.2	0.3 ^e^	0.4 ^e,f^	0.3	0.4	0.6 ^e,f^	0.5	0.5	0.7 ^e,f^	1.0	0.9	0.9
Dairy beverages	0.1	0.2 ^e^	0.2 ^f^	0.2	0.3 ^e^	0.4 ^e,f^	0.3	0.5 ^e^	0.5 ^f^	0.5	0.5	0.7 ^e,f^	0.7	0.7	0.8
Ice cream	0.0	0.0 ^e^	0.1 ^e,f^	0.0	0.1 ^e^	0.2 ^e,f^	0.2	0.2	0.4 ^e,f^	0.2	0.3	0.6 ^e,f^	0.5	0.7	0.8 ^f^
Ready-made sauces	0.1	0.1 ^e^	0.2 ^e,f^	0.2	0.3	0.5 ^e,f^	0.3	0.6 ^e^	0.8 ^e,f^	0.5	0.7 ^e^	1.0 ^e,f^	0.9	0.8	1.0 ^e,f^
Distilled alcoholic beverages	0.1	0.1	0.1	0.1	0.1	0.1	0.1	0.1	0.1	0.1	0.1	0.2 ^f^	0.2	0.2	0.2
Other ^d^	0.5	0.5	0.4 ^e,f^	0.3	0.4	0.4	0.3	0.4 ^e^	0.4 ^e,f^	0.4	0.4	0.5 ^f^	0.7	0.6	0.6

^a^ Other foods *in natura* or minimally processed, including teas and coffees, seafood, meat from other animals, nuts and seeds, and dried or dehydrated fruit and vegetables.

^b^ Other culinary ingredients including salt and other sugars.

^c^ Other processed foods, including dried and/or salted fish and seafood, canned cereals, legumes and vegetables, salted nuts, and canned diet/light meats.

^d^ Other ultra-processed foods, including reconstituted meats, ready-made tablets and seasonings, non-fat salt-based condiments, ultra-processed cheeses, and breakfast cereals.

^e^ p < 0.05 in the comparison with the previous period.

^f^ p < 0.05 for linear trend between 2002–2003 and 2017–2018.

When assessing the evolution of household food availability by income, one observes within the first four-fifths of income a clear trend towards a decrease in household availability foods *in natura* or minimally processed, and processed culinary ingredients, as opposed to an increase in ultra-processed foods between 2002–2003 and 2017–2018. On the other hand, in the top fifth of the income distribution, this trend is observed between 2002–2003 and 2008–2009, but stabilizes in the latest period. The second, third and fourth income fifths showed the largest increases in the percentages of share of ultra-processed foods.

Among foods *in natura* or minimally processed, an increase in the consumption of beef, eggs, and pork is observed in all fifths. In contrast, the consumption of cassava flour has decreased. Also, within this subgroup, we found an increase in the consumption of fruit and vegetables and a decrease in the consumption of beans from the first to the fourth income quintile. Among processed culinary ingredients, we observe an increase in starch consumption and a decrease in sugar consumption in all income quintiles. Among the ultra-processed foods there is a significant increase in cold cuts and sausages, cakes and sweet pies, sweets in general, pizza, lasagna or pastry, ready meals, ice cream and ready-made sauces in all income classes. Also notable is the increase in the purchase of ultra-processed breads, chocolate, non-carbonated sweetened beverages, and dairy drinks in the first four-fifths of income and of salted biscuits between the second-fifth of income and the last.

Table 3 shows the percentage share of food groups and subgroups, according to NOVA, in urban and rural areas between 2002–2003 and 2017–2018. Comparing urban and rural areas, in 2017–2018 the share of foods *in natura* or minimally processed was higher in rural than in urban areas (58.2% versus 47.1% of total calories), as was the share of processed culinary ingredients (24.5% versus 21.1%). On the other hand, the share of both processed and ultra-processed foods was higher in urban areas (11.1% and 20.6%, respectively) than in rural areas (5.8% and 11.5%).

**Table 3:** Relative share of groups and subgroups of the NOVA classification in the total calories determined by household food acquisition according to household situation, by year of the survey - Brazil - periods 2002–2003, 2008–2009 and 2017–2018.

Food groups and subgroups	Relative share by household situation (%)					
	Urban			Rural		
	2002–2003	2008–2009	2017–2018	2002–2003	2008–2009	2017–2018
**Foods in natura or minimally processed**	**49.1**	**47.2** ^e^	**47.1** ^f^	**61.9**	**58.3** ^e^	**58.2** ^f^
Rice	16.2	14.9 ^e^	14.4 ^f^	18.1	18.1	19.9
Milk	5.5	4.9 ^e^	4.8 ^f^	4.9	4.3	4.5
Poultry	3.2	3.6 ^e^	3.9 ^e,f^	2.3	3.2 ^e^	4.8 ^e,f^
Beans	5.0	4.3 ^e^	4.0 ^f^	8.2	5.9 ^e^	5.0 ^e,f^
Beef	3.5	4.3 ^e^	4.7 ^e,f^	2.4	4.1 ^e^	4.0 ^f^
Fruit	2.2	2.5 ^e^	2.9 ^e,f^	1.4	1.7	2.0 ^f^
Pasta	2.5	2.5	2.4	2.0	2.2	2.5 ^f^
Cornmeal	1.3	1.2	0.9 ^e,f^	3.6	2.7	2.3 ^f^
Cassava flour	2.7	2.0	1.3 ^e,f^	8.5	6.3	3.6 ^e,f^
Wheat flour	2.4	1.8	1.5 ^f^	3.8	2.8	2.7
Roots and tubers	1.1	1.1	1.3 ^e,f^	1.4	1.1	1.2
Eggs	0.3	0.7 ^e^	0.9 ^e,f^	0.4	0.7	1.0 ^e,f^
Vegetables	0.8	0.8	1.0 ^e,f^	0.5	0.6 ^e^	0.7 ^e,f^
Pork	0.7	0.6	0.9 ^e,f^	1.1	0.9	1.1
Fish	0.4	0.4	0.4	0.9	0.8	0.8
Corn, oats and other cereals	0.6	0.7	1.0 ^e,f^	1.5	1.8	1.4
Viscera	0.3	0.2	0.2	0.2	0.2	0.2
Outhers ^a^	0.5	0.6 ^e^	0.7 ^e,f^	0.6	0.8	0.6
**Processed culinary ingredients**	**25.2**	**22.8** ^e^	**21.1** ^e,f^	**26.8**	**26.5**	**24.5** ^e,f^
Vegetable oil	11.9	11.0 ^e^	10.7 ^f^	10.4	10.7	10.9
Sugar	12.2	10.8 ^e^	9.0 ^e,f^	14.2	14.3	12.3 ^e,f^
Animal fat	0.7	0.5 ^e^	0.7 ^e^	1.1	0.5 ^e^	0.5 ^f^
Starches	0.4	0.4	0.6 ^e,f^	0.7	0.8	0.7
Other ^b^	0.1	0.1	0.1	0.3	0.2	0.1 ^e,f^
**Processed foods**	**10.1**	**11.3** ^e^	**11.1**	**3.9**	**5.2** ^e^	**5.8** ^f^
Breads	7.4	8.2	7.5 ^e^	2.0	3.2 ^e^	3.9 ^e,f^
Cheese	1.0	1.2 ^e^	1.6 ^e,f^	0.5	0.5	0.5
Salted/dried/smoked meats	0.7	0.7	0.7	1.0	0.8	0.7 ^f^
Fermented alcoholic beverages	0.5	0.6 ^e^	0.7 ^e,f^	0.1	0.3 ^e^	0.3 ^f^
Other ^c^	0.5	0.5	0.6 ^e,f^	0.3	0.4	0.4
**Ultra-processed foods**	**15.6**	**18.7** ^e^	**20.6** ^e,f^	**7.4**	**10.0** ^e^	**11.5** ^e,f^
Cold cuts and sausages	2.1	2.6 ^e^	2.9 ^f^	1.0	1.6 ^e^	1.9 ^e,f^
Sweet cookies	2.0	2.2 ^e^	2.3 ^f^	1.3	1.5	1.6
Salted biscuits	1.4	1.6 ^e^	1.9 ^e,f^	1.4	1.6	2.0 ^e,f^
Margarine	1.9	2.1	1.9 ^e^	0.8	1.2 ^e^	1.2 ^f^
Cakes and sweet pies	0.8	1.2 ^e^	1.6 ^e,f^	0.3	0.5 ^e^	0.7 ^e,f^
Breads	1.0	1.3 ^e^	1.5 ^e,f^	0.5	0.5	0.5
Sweets in general	0.6	0.8 ^e^	1.0 ^e,f^	0.2	0.3 ^e^	0.4 ^f^
**Carbonated sweetened beverages**	1.8	1.8	1.3 ^e,f^	0.6	0.8 ^e^	0.6 ^e^
Chocolate	0.9	1.1 ^e^	1.3 ^f^	0.3	0.5 ^e^	0.6 ^f^
Pizza, lasagna or pastry	0.5	0.7 ^e^	0.9 ^e,f^	0.1	0.2 ^e^	0.4 ^e,f^
Ready meals	0.5	0.8 ^e^	1.0 ^e,f^	0.1	0.3 ^e^	0.4 ^e,f^
Non-carbonated sweetened beverages	0.5	0.5	0.6 ^e,f^	0.1	0.2 ^e^	0.3 ^f^
Dairy beverages	0.4	0.5 ^e^	0.6 ^e,f^	0.1	0.2 ^e^	0.2 ^e,f^
Ice cream	0.2	0.3 ^e^	0.5 ^e,f^	0.0	0.1 ^e^	0.1 ^e,f^
Ready-made sauces	0.4	0.5 ^e^	0.8 ^e,f^	0.1	0.2 ^e^	0.3 ^e,f^
Distilled alcoholic beverages	0.1	0.1	0.2	0.1	0.1	0.1
Other ^d^	0.4	0.5	0.5	0.3	0.3	0.3

^a^ Other foods *in natura* or minimally processed, including teas and coffees, seafood, meat from other animals, nuts and seeds, and dried or dehydrated fruit and vegetables.

^b^ Other culinary ingredients including salt and other sugars.

^c^ Other processed foods, including dried and/or salted fish and seafood, canned cereals, legumes and vegetables, salted nuts, and canned diet/light meats.

^d^ Other ultra-processed foods, including reconstituted meats, ready-made tablets and seasonings, non-fat salt-based condiments, ultra-processed cheeses, and breakfast cereals.

^e^ p < 0.05 in the comparison with the previous period.

^f^ p < 0.05 for linear trend between 2002–2003 and 2017–2018.

Consumption trends in urban and rural areas follow national trends, with a decline in consumption of foods *in natura* or minimally processed and processed culinary ingredients observed between 2002–2003 and 2017–2018, at the expense of an increase in consumption of ultra-processed foods. In the rural area a positive and significant trend was also observed in the percentage share of processed foods.

Table 4 describes the availability of food groups and subgroups, according to the major regions of the country. In 2017–2018 the share of foods *in natura* or minimally processed in total caloric food availability was the highest in the North and Northeast (58.3% and 54.2%, respectively) and the lowest in the Southeast, South, and Midwest (44.3%, 46.2%, and 50.5%). The shares of processed culinary ingredients and processed foods in total calories showed smaller variations, standing at 19.9%–23.9% and 8.3%–11.6%, respectively, in all regions. The share of ultra-processed foods in total calorie availability was the highest in the South, Southeast, and Midwest (23.5%, 22.5%, and 17.3%) and the lowest in the North and Northeast (11.9%, and 14.3%).

**Table 4:** Relative share of groups and subgroups of the NOVA classification in the total calories determined by household food acquisition according to the large regions of Brazil, by year of the survey - Brazil - periods 2002–2003, 2008–2009 and 2017–2018.

Food groups and subgroups	Relative share according to region of residence (%)														
	North			Northeast			Southeast			South			Midwest		
	2002/03	2008/09	2017/18	2002/03	2008/09	2017/18	2002/03	2008/09	2017/18	2002/03	2008/09	2017/18	2002/03	2008/09	2017/18
**Foods in natura or minimally processed**	**62.4**	**58.4** ^e^	**58.3** ^f^	**56.1**	**53.8**	**54.2**	**46.2**	**44.4** ^e^	**44.3** ^f^	**51.2**	**47.7** ^e^	**46.2** ^f^	**52.5**	**52.0**	**50.5**
Rice	17.1	15.5	18.3	16.0	15.1	15.8	16.9	15.5	14.9 ^f^	12.5	11.9	11.1	24.7	22.8	19.3 ^e,f^
Milk	3.8	3.4	3.8	4.2	3.9 ^e^	4.3 ^e^	5.9	5.3 ^e^	5.0 ^f^	6.2	5.8	5.4 ^f^	5.8	4.4 ^e^	4.4 ^f^
Poultry	3.8	4.8 ^e^	6.1 ^e,f^	2.9	4.3 ^e^	5.4 ^e,f^	3.2	3.2	3.1	3.1	3.2	3.6 ^e,f^	2.8	2.9	4.0 ^e,f^
Beans	4.5	4.4	3.7 ^e,f^	8.3	6.3 ^e^	5.5 ^e,f^	4.8	4.1	4.0 ^f^	3.9	2.9 ^e^	2.8 ^f^	4.8	4.4	3.7 ^e,f^
Beef	4.4	5.4 ^e^	6.2 ^e,f^	3.0	4.1 ^e^	4.5 ^f^	3.0	3.6 ^e^	3.9 ^f^	4.2	5.4 ^e^	5.1 ^f^	3.9	5.6 ^e^	6.1 ^f^
Fruit	2.8	2.5	2.8	1.6	2.3	2.6 ^e,f^	2.1	2.5	2.9 ^e,f^	2.5	2.5	2.9 ^e^	1.5	2.1 ^e^	2.8 ^e,f^
Pasta	1.7	2.1	1.8	2.7	2.6	2.8	2.4	2.4	2.3	2.5	2.6	2.6	1.8	1.8	1.6 ^e,f^
Cornmeal	0.8	1.0	0.9	3.5	3.0 ^e^	2.1 ^e,f^	1.0	0.9	0.7 ^e,f^	1.3	0.9 ^e^	0.8 ^f^	0.6	0.7	0.5
Cassava flour	15.4	11.5	7.6 ^e,f^	8.1	5.5 ^e^	3.0 ^e,f^	0.8	0.7	0.5 ^e,f^	0.5	0.4	0.3 ^e,f^	0.8	0.8	0.5 ^e,f^
Wheat flour	1.1	1.1	1.1	0.9	0.6	0.7	1.9	1.5	1.3 ^f^	8.3	5.9 ^e^	4.9 ^f^	2.1	1.8	1.4 ^f^
Roots and tubers	0.8	0.7	0.5 ^f^	0.9	0.9 ^e^	1.2 ^e,f^	1.1	1.1	1.2 ^e^	1.7	1.7	1.7	0.8	1.0 ^e^	1.3 ^e,f^
Eggs	0.5	0.6 ^e^	0.8 ^f^	0.4	0.7	1.1 ^e,f^	0.0	0.7 ^e^	0.9 ^e,f^	0.9	0.8	1.0 ^e^	0.4	0.5 ^e^	0.9 ^e,f^
Vegetables	0.5	0.6	0.6	0.7	0.7	0.9 ^e,f^	0.8	0.9	1.0 ^e,f^	0.7	0.8 ^e^	0.9 ^e,f^	0.7	0.8 ^e^	1.0 ^e,f^
Pork	0.6	0.4	0.4	0.4	0.3	0.6 ^e,f^	0.7	0.7	1.1 ^e,f^	1.5	1.1 ^e^	1.3	0.8	0.6	1.3 ^e,f^
Fish	2.8	2.2	1.8 ^f^	0.7	0.7 ^e^	0.6	0.3	0.3	0.2 ^e^	0.2	0.2	0.2	0.2	0.2	0.3 ^f^
Corn, oats and other cereals	0.5	0.8 ^e^	0.8 ^f^	1.0	1.5	2.0 ^e,f^	0.7	0.5	0.6	0.5	0.7	0.8	0.4	1.0 ^e^	0.9
Viscera	0.4	0.3	0.3	0.3	0.3 ^e^	0.3	0.2	0.2	0.2	0.2	0.2	0.3	0.2	0.2	0.2
Others ^a^	0.9	1.0	0.7	0.6	0.8 ^e^	0.8 ^f^	0.4	0.4	0.6 ^e,f^	0.5	0.8 ^e^	0.6	0.3	0.5 ^e^	0.6 ^f^
**Processed culinary ingredients**	**22.2**	**22.4**	**21.2**	**23.8**	**22.1**	**19.9** ^e,f^	**26.6**	**24.2** ^e^	**22.3** ^e,f^	**24.2**	**22.4** ^e^	**21.4** ^f^	**30.4**	**26.4** ^e^	**23.9** ^e,f^
Vegetable oil	10.1	10.0	9.9	8.7	8.4 ^e^	7.8 ^e,f^	13.0	12.1	12.0	11.3	11.4	11.5	16.2	13.9 ^e^	13.3 ^f^
Sugar	10.5	11.0	9.5 ^e,f^	13.6	12.2	10.3 ^e,f^	12.6	11.4	9.3 ^e,f^	10.9	9.7	8.7 ^f^	13.1	11.5 ^e^	8.8 ^e,f^
Animal fat	0.6	0.5	0.5	0.4	0.5	0.6 ^e,f^	0.7	0.5 ^e^	0.7 ^e^	1.6	0.8 ^e^	0.8 ^f^	0.7	0.5	1.0
Starches	0.9	0.9	1.2	0.8	0.9	1.1 ^e,f^	0.2	0.3	0.4 ^f^	0.2	0.2	0.4 ^e,f^	0.4	0.5	0.7 ^e,f^
Other ^b^	0.1	0.1	0.0	0.2	0.2 ^e^	0.1 ^e,f^	0.0	0.1	0.0	0.2	0.1	0.1	0.1	0.1	0.2
**Processed foods**	**7.2**	**7.9**	**8.6**	**9.0**	**10.9** ^e^	**11.6** ^f^	**10.6**	**11.5**	**10.9**	**7.4**	**8.6** ^e^	**8.9**	**6.8**	**7.6**	**8.3** ^f^
Breads	5.9	6.3	6.7	6.6	8.2 ^e^	8.9 ^f^	7.6	8.2	6.9 ^e^	4.6	5.3	4.9 ^f^	4.8	5.3	5.0
Cheese	0.3	0.4	0.5	0.5	0.9	1.0 ^f^	1.2	1.3	1.8 ^e,f^	1.2	1.5	1.7 ^f^	0.7	0.8	1.3 ^e,f^
Salted/dried/smoked meats	0.6	0.6	0.7	1.2	1.1	0.9 ^f^	0.7	0.7	0.7	0.3	0.4	0.4	0.4	0.4	0.6 ^e,f^
Fermented alcoholic beverages	0.2	0.3	0.3	0.2	0.3	0.3	0.5	0.7 ^e^	0.8 ^f^	0.6	0.8	1.1 ^e,f^	0.5	0.6	0.8 ^e,f^
Other ^c^	0.3	0.4 ^e^	0.4	0.4	0.5 ^e^	0.5	0.6	0.5	0.6	0.6	0.7	0.8 ^f^	0.4	0.5	0.5
**Ultra-processed foods**	**8.2**	**11.3** ^e^	**11.9** ^f^	**11.2**	**13.2** ^e^	**14.3** ^f^	**16.6**	**19.8** ^e^	**22.5** ^e,f^	**17.2**	**21.4** ^e^	**23.5** ^e,f^	**10.3**	**13.9** ^e^	**17.3** ^e,f^
Cold cuts and sausages	1.0	1.4 ^e^	1.8 ^f^	1.1	1.4	1.9 ^e,f^	2.6	3.1 ^e^	3.3 ^f^	2.4	3.0 ^e^	3.3 ^e,f^	1.3	1.9 ^e^	2.3 ^e,f^
Sweet cookies	0.8	1.1 ^e^	1.1	1.9	2.0	2.2	2.1	2.3	2.3	2.1	2.2	2.3	1.4	1.6	1.9 ^f^
Salted biscuits	1.4	1.7 ^e^	1.6	2.4	2.3	2.5	1.0	1.3 ^e^	1.8 ^e,f^	1.1	1.3	1.4	0.7	1.1 ^e^	1.5 ^e,f^
Margarine	1.4	1.6	1.5	2.0	2.1 ^e^	1.9	1.7	2.0	1.9	1.8	1.9	1.7	1.3	1.5	1.3 ^e^
Cakes and sweet pies	0.3	0.6 ^e^	0.6 ^f^	0.5	0.8 ^e^	1.1 ^e,f^	0.8	1.2 ^e^	1.7 ^e,f^	1.0	1.8 ^e^	1.8 ^f^	0.5	0.7 ^e^	1.4 ^e,f^
Breads	0.5	0.8 ^e^	1.0 ^e,f^	0.3	0.5 ^e^	0.5 ^f^	1.0	1.3 ^e^	1.7 ^e,f^	1.9	2.0	2.2	0.6	1.0 ^e^	1.4 ^e,f^
Sweets in general	0.2	0.4 ^e^	0.6 ^e,f^	0.2	0.4	0.4 ^f^	0.7	0.9 ^e^	1.1 ^e,f^	0.7	1.0 ^e^	1.3 ^e,f^	0.4	0.7 ^e^	0.9 ^e,f^
Carbonated sweetened beverages	0.9	1.2 ^e^	0.7 ^e,f^	0.8	0.9 ^e^	0.7 ^e,f^	2.0	2.0	1.4 ^e,f^	1.9	2.0	1.7 ^e,f^	1.5	1.6	1.2 ^e,f^
Chocolate	0.3	0.6 ^e^	0.5 ^f^	0.3	0.4 ^e^	0.5 ^f^	1.1	1.3	1.5 ^f^	1.2	1.6 ^e^	1.8 ^f^	0.6	0.9 ^e^	1.0 ^e,f^
Pizza, lasagna or pastry	0.1	0.3 ^e^	0.4 ^e,f^	0.2	0.3 ^e^	0.4 ^e,f^	0.6	0.8 ^e^	1.1 ^e,f^	0.6	0.9 ^e^	1.1 ^e,f^	0.3	0.5	0.9 ^e,f^
Ready meals	0.2	0.3 ^e^	0.5 ^e,f^	0.2	0.3 ^e^	0.4 ^e,f^	0.6	0.9 ^e^	1.2 ^e,f^	0.5	1.0 ^e^	1.2 ^e,f^	0.3	0.6 ^e^	1.0 ^e,f^
Non-carbonated sweetened beverages	0.2	0.4 ^e^	0.2 ^e^	0.1	0.2 ^e^	0.2 ^f^	0.6	0.6	0.7 ^e^	0.4	0.6 ^e^	0.8 ^e,f^	0.3	0.5 ^e^	0.6 ^e,f^
Dairy beverages	0.1	0.3 ^e^	0.3 ^f^	0.2	0.4 ^e^	0.4 ^f^	0.5	0.5	0.6 ^e,f^	0.4	0.6 ^e^	0.7 ^e,f^	0.2	0.4 ^e^	0.4 ^f^
Ice cream	0.0	0.1 ^e^	0.2 ^e,f^	0.1	0.1	0.2 ^e,f^	0.3	0.4	0.6 ^e,f^	0.2	0.4 ^e^	0.6 ^e,f^	0.2	0.2	0.5 ^e,f^
Ready-made sauces	0.2	0.2 ^e^	0.4 ^e,f^	0.2	0.2	0.4 ^e,f^	0.5	0.6	0.9 ^e,f^	0.6	0.8 ^e^	1.1 ^e,f^	0.3	0.4 ^e^	0.6 ^e,f^
Distilled alcoholic beverages	0.0	0.1 ^e^	0.2	0.1	0.1	0.1	0.1	0.1	0.2	0.2	0.1	0.2 ^e^	0.1	0.1 ^e^	0.1
Other ^d^	0.3	0.3	0.3	0.6	0.7	0.5 ^e,f^	0.4	0.4	0.5 ^e^	0.2	0.3 ^e^	0.4 ^e,f^	0.2	0.3	0.4 ^f^

^a^ Other foods *in natura* or minimally processed, including teas and coffees, seafood, meat from other animals, nuts and seeds, and dried or dehydrated fruit and vegetables.

^b^ Other culinary ingredients including salt and other sugars.

^c^ Other processed foods, including dried and/or salted fish and seafood, canned cereals, legumes and vegetables, salted nuts, and canned diet/light meats.

^d^ Other ultra-processed foods, including reconstituted meats, ready-made tablets and seasonings, non-fat salt-based condiments, ultra-processed cheeses, and breakfast cereals.

^e^ p < 0.05 in the comparison with the previous period.

^f^ p < 0.05 for linear trend between 2003 and 2018.

When assessing the trend of food procurement between 2002–2003 and 2017–2018, according to major regions, a significant drop in the purchase of foods *in natura* or minimally processed was observed in the North, Southeast, and South. Among the foods in this group we found a drop in the consumption of beans and cassava flour in all regions, in contrast to the increase in beef. Consumption of processed culinary ingredients declined in all regions except the North, whose share remained relatively stable between 2002–2003 and 2017–2018. In all regions there was a significant decline in the share of sugar in purchases. The consumption of processed foods remained stable in the North, Southeast, and South, and increased in the Northeast and Midwest regions. The increase in ultra-processed food purchases was observed in all regions. Trends in the purchase of carbonated sweetened beverages over the period evaluated proved quite similar in the five regions, with increase in their consumption between 2002–2003 and 2008–2009 followed by a decrease between 2008–2009 and 2017–2018.

Table 5 describes the evolution of household availability of food groups and subgroups based on the POFs of the country’s metropolitan regions conducted in 1987–1988, 1995–1996, 2002–2003, 2008–2009, and 2017–2018. Over this long period, we observe a decline in the share of foods *in natura* or minimally processed and processed culinary ingredients, and an increase in the relative percentage of processed and ultra-processed foods. In 1987–1988 the calorie share of the sum of the group of foods *in natura* or minimally processed and processed culinary ingredients made up about 80% of the calories consumed, while ultra-processed foods contributed only 10% of the calorie share. The period of the greatest increase in the share of ultra-processed foods and consequent decrease in foods *in natura* or minimally processed and culinary ingredients occurred between 1995 and 2003, when the annual growth rate of the share of ultra-processed foods was 0.8%, while in the other periods the growth rate observed was close to 0.2 to 0.3 percentage points per year. After 30 years, in 2017, the sum of the share of foods *in natura* or minimally processed and culinary ingredients accounted for 64% of dietary calories, while ultra-processed foods accounted for about 24% of them.

**Table 5:** Relative share of foods and food groups of the NOVA classification in total calories determined by household food acquisition in metropolitan regions - periods 1987–1988, 1995–1996, 2002–2003, 2008–2009 e 2017–2018.

Food groups and subgroups	Relative share, per survey year (%)				
	1987–1988	1995–1996	2002–2003	2008–2009	2017–2018
**Foods in natura or minimally processed**	**51.5**	**50.9**	**45.8**	**44.7**	**44.9** ^f^
Rice	15.8	15.6	13.9	14.2	12.2 ^f^
Milk	6.5	6.8	6.0	5.2	5.2 ^f^
Poultry	3.6	4.9	4.2	3.7	4.0
Beans	5.3	5.0	5.0	4.5	4.2 ^f^
Beef	3.2	4.0	3.4	3.5	5.0 ^f^
Fruit	2.7	2.5	2.4	2.6	3.2 ^f^
Pasta	2.2	2.2	2.7	2.7	2.9 ^f^
Cornmeal	1.2	1.1	0.9	1.0	0.8 ^f^
Cassava flour	2.7	2.2	2.0	1.5	1.2 ^f^
Wheat flour	2.2	1.8	1.6	1.4	1.3 ^f^
Roots and tubers	1.4	1.1	1.2	1.1	1.3
Eggs	1.5	1.1	0.2	0.9	1.0 ^f^
Vegetables	1.0	0.9	0.8	0.8	1.0
Pork	0.8	0.5	0.4	0.4	0.7
Fish	0.4	0.4	0.4	0.4	0.3
Corn, oats and other cereals	0.1	0.2	0.3	0.2	0.3 ^f^
Viscera	0.8	0.4	0.4	0.3	0.3 ^f^
Other ^a^	0.1	0.1	0.2	0.2	0.2 ^f^
**Processed culinary ingredients**	**27.3**	**25.3**	**23.1**	**21.4**	**19.4** ^f^
Vegetable oil	12.3	11.0	11.1	9.8	9.9 ^f^
Sugar	13.3	13.0	10.6	10.0	7.7 ^f^
Animal fat	1.1	0.8	1.0	1.2	1.1
Starches	0.6	0.4	0.3	0.2	0.6
Other ^b^	0.1	0.0	0.1	0.1	0.1 ^f^
**Processed foods**	**11.1**	**11.9**	**12.9**	**13.2**	**12.1** ^f^
Breads	9.2	9.7	10.0	10.3	8.1
Cheese	0.9	1.1	1.3	1.4	2.0 ^f^
Salted/dried/smoked meats	0.4	0.4	0.8	0.7	0.8 ^f^
Fermented alcoholic beverages	0.3	0.5	0.5	0.6	0.8 ^f^
Other ^c^	0.3	0.2	0.2	0.2	0.4 ^f^
**Ultra-processed foods**	**10.2**	**12.0**	**18.2**	**20.7**	**23.7** ^f^
Cold cuts and sausages	0.7	1.7	2.5	2.8	2.9 ^f^
Sweet cookies	1.1	1.6	2.2	2.2	2.4 ^f^
Salted biscuits	0.9	1.2	1.3	1.5	1.7 ^f^
Margarine	2.0	1.5	2.3	2.4	2.2 ^f^
Cakes and sweet pies	0.6	0.6	0.4	1.1	1.7 ^f^
Breads	0.6	0.4	1.0	1.2	2.0 ^f^
Sweets in general	1.2	0.7	0.9	1.2	1.6 ^f^
Carbonated sweetened beverages	0.8	1.2	2.0	2.0	1.4 ^f^
Chocolate	0.4	0.4	0.9	0.9	1.2 ^f^
Pizza, lasagna or pastry	0.2	0.4	0.5	0.5	0.8 ^f^
Ready meals	0.4	0.8	1.0	1.3	1.8 ^f^
Non-carbonated sweetened beverages	0.2	0.4	1.3	1.6	0.9 ^f^
Dairy beverages	0.1	0.1	0.5	0.5	0.6 ^f^
Ice cream	0.1	0.2	0.3	0.3	0.6 ^f^
Ready-made sauces	0.2	0.2	0.4	0.4	0.5 ^f^
Distilled alcoholic beverages	0.2	0.1	0.1	0.1	0.2
Other ^d^	0.4	0.4	0.5	0.6	1.2 ^f^

^a^ Other foods *in natura* or minimally processed, including teas and coffees, seafood, meat from other animals, nuts and seeds, and dried or dehydrated fruit and vegetables.

^b^ Other culinary ingredients including salt and other sugars.

^c^ Other processed foods, including dried and/or salted fish and seafood, canned cereals, legumes and vegetables, salted nuts, and canned diet/light meats.

^d^ Other ultra-processed foods, including reconstituted meats, ready-made tablets and seasonings, non-fat salt-based condiments, ultra-processed cheeses, and breakfast cereals.

^f^ p < 0.05 for linear trend between 2002–2003 and 2017–2018.

## DISCUSSION

The results of this study show that foods *in natura* or minimally processed and culinary ingredients, the basis of culinary preparations traditionally consumed in the country, still predominate in the diet of the Brazilian population. In the North, Midwest, and Northeast regions, in rural areas and among families with lower incomes, the share of foods *in natura* or minimally processed and culinary ingredients contribute more than 50% of the calories purchased daily. In the South and Southeast regions, in metropolitan and urban areas, and among families with higher incomes, although foods *in natura* or minimally processed and processed culinary ingredients still predominate, ultra-processed foods already represent more than a fifth of the calories purchased by households.

Our results further reinforce the trend of increasing share of ultra-processed foods at the expense of consumption of culinary preparations ^11^ . But they also indicate a deceleration of this increase, which was 0.4 pp/year between 2002 and 2009 and went down to 0.2 pp/year between 2008 and 2018. We found similar trends in urban and rural households, across all regions and income levels in the country. In the metropolitan regions, for the period of about 30 years, the relative share of ultra-processed foods in the diet increased from 10.2% to 23.7% of total calories, representing an increase of 13.5% (more than 130% increase), with the interval from 1995 to 2003 showing the highest growth rate, 0.8% per year. Following the country’s trend, the growth speed of this food group has also decreased in the most recent period.

Opposite to foods *in natura* or minimally processed, we observed an increase in the availability of fruit and beef in the population’s diet between 2002–2003 and 2017–2019. Fruit consumption is considered a marker of healthy eating, as it is associated with protection against excessive weight gain and the development of several chronic non-communicable diseases ^12^ . Even if this increase is seen as a positive aspect in the eating pattern, the consumption of fruit by the Brazilian population (approximately 54.9 g/day - non-tabulated data) is still far below the recommendation of the World Health Organization (400 g/day ^12^ . Regarding beef, although it belongs to the group of foods *in natura* or minimally processed, its excessive consumption is associated with the development of diseases, such as certain types of cancers, cardiovascular diseases, and others ^15^ , besides resulting in a high environmental impact ^19^ . It is worth noting that the increase in the share of beef in the diet of the population is observed in all income fifths, with the highest intensity in the lowest income fifths (the one with the lowest consumption at the beginning of the period studied).

The ultra-processed food subgroups generally showed similar trends, increasing between 1987–1988 and 2017–2018 in metropolitan regions and between 2002–2003 and 2017–2018 nationwide. The increase in the purchase of cold cuts and sausages, cakes and sweet pies, salted biscuits, ultra-processed breads, and ready meals stands out. Among the subgroups of ultra-processed foods, the exception was the subgroup of carbonated sweetened beverages that presented an increase in the first periods (1987–1988 to 2002–2003), observed only for the metropolitan regions for the longest time series under analysis, followed by stability and fall, both for metropolitan regions and for the country. The fall was more accentuated among households with greater economic power.

The decrease in consumption of carbonated sweetened beverages found in this study has also been observed in capital cities by the *Sistema de Monitoramento de Fatores de Risco e Proteção para Doenças Crônicas* (Risk and Protective Factor Monitoring System for Chronic Diseases) ^20^ as a result of a likely awareness of its harmful effects. The health risks associated with consumption of sweetened beverages, especially soft drinks, are widely pointed out in the literature ^21^ , whose recommendation is to reduce the consumption of soft drinks and other sweetened beverages, according to the Pan American Health Organization ^25^ and the World Health Organization ^26 , 27^ .

The increase in the consumption of the other subgroups of ultra-processed foods, even if with a decrease in the speed of growth, remains worrying. The results show that this consumption is higher in the households with higher income, in the more developed regions, South and Southeast (22.5% and 23.5%), in the urban area (28.6%), and in the metropolitan regions (23.7%). There is a robust body of evidence in the scientific literature associating the consumption of this group of foods with poorer diet quality, with a higher percentage of free sugar, total and saturated fat, lower concentration of fiber and protein, and lower content of several minerals and vitamins ^28^ . In addition to diet quality, evidence associates the greater share of this group of foods with increased risk of weight gain and obesity, diabetes, and cardiovascular disease, among others ^2 , 5^ .

Despite the increasing trend, the share of ultra-processed food in Brazil is still lower, when compared to countries with higher income *per capita* , such as the USA, Canada, the UK, and Australia ^29^ , and in other middle-income countries, such as Chile and Mexico ^33 , 34^ . Brazil has a rich and diverse food culture, and each region has traditional regional food preparations ^35^ , which could explain, at least in part, the predominance of culinary preparations in the Brazilian dietary pattern. Another possible explanation for this is the relative prices of these foods in the country. In Brazil, the food pattern based on ultra-processed foods is still more expensive than that based on foods *in natura* or minimally processed ^36^ . However, projection analyses indicate a tendency to reverse this incentive, due to the constant relative reduction in the prices of ultra-processed foods and the increase in the price of fresh foods, observed since the beginning of the 2000s ^37^ . It is natural to believe that this trend has an influence on the increase in the share of ultra-processed foods observed in the period. It should also be kept in mind that, parallel to the scenario of change in prices, from 2014 on, the country experienced a significant economic crisis, with a period of inflationary pressure and income reduction for a significant portion of the population ^38 , 39^ .

Educational actions may also have significantly influenced this process, especially since the publication of the *Guia Alimentar para a População Brasileira* (Food Guide for the Brazilian Population), in 2014 ^4^ . The Guide was a pioneer in considering the degree of food processing in its recommendations, with direct orientations to avoid the consumption of ultra-processed foods. The document started a movement to raise awareness among health professionals and the population about the harmful effects of ultra-processed food consumption, serving as the basis for the creation of public policies aimed at reducing it. One example is the publication of resolution n.6 of May 8, 2020, which brought greater alignment of the *Programa Nacional de Alimentação Escolar com o Guia Alimentar* (National School Feeding Program with the Food Guide), limiting the acquisition of processed and ultra-processed foods with program resources to a maximum of 20%, and prohibiting the supply of ultra-processed foods for children up to three years old ^40^ .

Despite these advances and the slowing down of the growth of ultra-processed food in the Brazilian diet, the actions and policies implemented so far have failed to contain the general increase of this group, especially among lower income fifths. Promoting adequate and healthy eating implies the engagement and articulation of different sectors and players that need to advance in measures that promote healthy environments in institutional spaces, such as the regulation of ultra-processed food sales in school canteens; protection measures, such as the regulation of ultra-processed food advertising, especially that targeted at children; and pricing and taxation policies. Countries such as Mexico, France, the UK, and Hungary, for example, have adopted policies of taxing sweetened drinks ^41^ and the first results in Mexico, for example, showed a 6% reduction in the purchase of such drinks ^45^ . Similar policies could be incorporated and expanded to other ultra-processed foods in Brazil.

It is important to mention that changes in the political context in the last few years put at risk the advances achieved so far. In a technical note published in September 2020, the Ministry of Agriculture, Livestock, and Supply claimed a revision of the *Guia Alimentar para a População Brasileira* (Food Guide for the Brazilian Population) under the false pretense that there was no scientific evidence to support the recommendations and guidelines of the Guide ^46^ . The published note caused repudiation from organizations and academic institutions from Brazil and other countries that manifested in defense of the Brazilian Food Guide. A growing number of scientific studies highlight the association between ultra-processed foods and a decline in quality of diet and health outcomes ^2 , 5^ . Other countries, such as France, Canada, and Uruguay, have adopted targets to decrease the consumption of ultra-processed foods in their public policies ^47^ . This exposes the fragility of food and nutrition policies in Brazil, resulting from economic pressures.

## CONCLUSIONS

This study stands out for evaluating the availability of food in Brazilian households considering the groups and subgroups of the NOVA classification, which is internationally recognized and used in the recommendations of food guides. Although the study evaluates the diet of Brazilians based only on the availability of food for consumption at home and not its effective consumption by individuals, these data are useful for monitoring the feeding pattern of the Brazilian population, especially when the indicators used focus on the relative share and not the absolute quantities of food, and when eating at home represents about 70% of the calories ingested by the population ^50^ . Even with limitations, family purchases are related to individual consumption patterns ^51^ and, in Brazil, these data are the only source old enough to allow an analysis of food trends in the population, since data on actual consumption for a representative sample of the population are only available from 2008–2009 ^52^ . The short reference period (one week) for data collection on household food purchases means that POF estimates must be calculated from aggregates of households and not from individual households, as done in this study.

Among the strengths of this study, the following stand out: the rigorously probabilistic nature of the survey sample and the representativeness for the metropolitan regions from 1987 to 2017 and for the country as of 2003; the distribution of the sampling among the 12 months of the year, allowing for seasonal variations in food consumption.

Finally, we highlight that the trends in the dietary pattern of the Brazilian population, with the increasing share of ultra-processed foods revealed by this study, are consistent with the growing share of chronic non-communicable diseases in the morbidity and mortality profile of the Brazilian population, and particularly with the increasing prevalence of overweight and obesity in the country.

## Footnotes

a Instituto Brasileiro de Geografia e Estatística, Diretoria de Pesquisas, Coordenação de Trabalho e Rendimento. Pesquisa de Orçamentos Familiares 2017-2018: avaliação nutricional da disponibilidade domiciliar de alimentos no Brasil. Rio de Janeiro: IBGE; 2020.

b IBGE. SIDRA: banco de dados agregados. Brasília: Instituto Brasileiro de Geografia e Estatística; 2012.

c IBGE. Tabela de composição de alimentos. Rio de Janeiro: Instituto Brasileiro de Geografia e Estatística; 1969.

d Universidade de São Paulo, Food Research Center. TBCA - Tabela Brasileira de Composição de Alimentos. São Paulo: FORC; 2019.
