# Supplementary material for: Three decades of household food availability according to NOVA - Brazil, 1987–2018
Source: Rev Saude Publica. 2022 Aug 1;56:75. doi: 10.11606/s1518-8787.2022056004570 (PMC9388064; doi:10.11606/s1518-8787.2022056004570)
Supplement: Translation

# Translation: Três décadas da disponibilidade domiciliar de alimentos segundo a NOVA – Brasil, 1987–2018

**Authors:** Renata Bertazzi Levy, Giovanna Calixto Andrade, Gabriela Lopes da Cruz, Fernanda Rauber, Maria Laura da Costa Louzada, Rafael Moreira Claro, Carlos Augusto Monteiro

**Affiliations:** I Brasil Universidade de São Paulo. Faculdade de Medicina. Departamento de Medicina Preventiva. São Paulo, SP, Brasil; II Brasil Universidade de São Paulo. Núcleo de Pesquisas Epidemiológicas em Nutrição e Saúde. São Paulo, SP, Brasil; III Brasil Universidade de São Paulo. Faculdade de Saúde Pública. Programa de Pós-Graduação em Nutrição em Saúde Pública. São Paulo, SP, Brasil; IV Brasil Universidade de São Paulo. Faculdade de Saúde Pública. Departamento de Nutrição. São Paulo, SP, Brasil; V Brasil Universidade Federal de Minas Gerais. Escola de Enfermagem. Departamento de Nutrição. Belo Horizonte, MG, Brasil

**Keywords:** Alimentos, Dieta e Nutrição, Alimentação Básica, Alimentos Industrializados, Fatores Socioeconômicos, Economia dos Alimentos

## Abstract

**OBJETIVO:**

Avaliar a tendência da aquisição domiciliar de alimentos de acordo com a classificação NOVA no Brasil entre 1987–1988 e 2017–2018.

**MÉTODOS:**

Foram utilizados dados de aquisição domiciliar de alimentos provenientes de cinco edições da Pesquisas de Orçamentos Familiares, realizada pelo Instituto Brasileiro de Geografia e Estatística, nos anos 1987–1988, 1995–1996, 2002–2003, 2008–2009 e 2017–2018. Todos os alimentos reportados foram categorizados segundo a classificação NOVA. A disponibilidade domiciliar dos grupos e subgrupos de alimentos foi expressa por meio de sua participação (%) nas calorias totais, para o conjunto das famílias brasileiras, por situação do domicílio (urbana ou rural), para cada uma das cinco regiões geográficas do país, por quintos da distribuição de renda domiciliar *per capita* (inquéritos de 2002–2003, 2008–2009 e 2017–2018); e para as 11 principais regiões urbanas do país (inquéritos de 1987–1988, 1995–1996, 2002–2003, 2008–2009 e 2017–2018). Modelos de regressão linear foram utilizados para avaliar a tendência de aumento ou diminuição na aquisição dos alimentos.

**RESULTADOS:**

A dieta da população brasileira ainda é composta predominantemente por alimentos *in natura* e minimamente processados e ingredientes culinários processados. No entanto, nossos achados apontam tendências de aumento da participação de alimentos ultraprocessados na dieta. Esse aumento que foi de 0,4 pontos percentuais ao ano na primeira porção do período estudado, entre 2002 e 2009, e desacelerou para 0,2 pontos percentuais entre 2008 e 2018. O consumo de alimentos ultraprocessados foi maior entres os domicílios de maior renda, nas regiões Sul e Sudeste, na área urbana, e nas regiões metropolitanas.

**CONCLUSÃO:**

Os resultados do presente estudo apontam um aumento na participação de alimentos ultraprocessados na dieta dos brasileiros. Cenário preocupante, uma vez que o consumo de tais alimentos está associado ao desenvolvimento de doenças e à perda da qualidade nutricional da dieta.

## INTRODUÇÃO

Diversas entidades internacionais, como a Organização Mundial da Saúde (OMS) e a Organização das Nações Unidas para a Alimentação (FAO), defendem que padrões alimentares saudáveis e sustentáveis são aqueles baseados em uma grande variedade de alimentos *in natura* ou minimamente processados e restritos em alimentos altamente processados ^1^ . Embora haja mais de uma classificação que agrupa os alimentos conforme o processamento a que foram submetidos, o sistema NOVA é, de longe, o mais utilizado ^2^ , segundo o qual, os alimentos são classificados de acordo com a extensão e o propósito do seu processamento industrial, em quatro grandes grupos: alimentos *in natura* ou minimamente processados, ingredientes culinários processados, alimentos processados e alimentos ultraprocessados ^3^ . Com base nessa classificação, o *Guia Alimentar para a População Brasileira* , publicado em 2014, recomenda: que a alimentação seja baseada em larga variedade de alimentos *in natura* ou minimamente processados; que ingredientes culinários processados sejam utilizados em pequena quantidade para transformar alimentos do primeiro grupo em preparações culinárias; que alimentos processados, também em pequena quantidade, sejam utilizados como parte de preparações culinárias ou acompanhamento; e que o consumo de alimentos ultraprocessados seja evitado ^4^ .

Inúmeras evidências, incluindo revisões sistemáticas de estudos de coorte e com meta-análises, mostram que o maior consumo de alimentos ultraprocessados está associado ao risco de doenças crônicas não transmissíveis, como obesidade, hipertensão, diabetes, dislipidemias, doenças cardiovasculares, depressão, cânceres – como o de mama –, distúrbios gastrointestinais, bem como mortalidade precoce por todas as causas ^2 , 5^ .

No Brasil, a evolução da disponibilidade domiciliar de alimentos classificados segundo o sistema NOVA vem sendo documentada com base em Pesquisas de Orçamentos Familiares (POFs), realizadas pelos Instituto Brasileiro de Geografia e Estatística (IBGE) desde 1987–1988, nas áreas metropolitanas, e desde 2002–2003, no país como um todo. A participação de ultraprocessados na aquisição domiciliar de alimentos aumentou tanto nas áreas metropolitanas entre 1987–1988 e 2002–2003, quanto para o conjunto do país entre 2002–2003 e 2008–2009, observando-se importantes variações, segundo localização e nível de renda do domicílio ^11^ .

A realização de nova POF pelo IBGE em 2017–2018 permite a atualização da tendência de evolução no Brasil da aquisição domiciliar de alimentos, classificados de acordo com a extensão e o propósito do seu processamento, que é apresentada a seguir neste artigo.

## MÉTODOS

O presente estudo utilizou dados de aquisição domiciliar de alimentos provenientes de cinco edições da POF, realizada pelo IBGE, nos períodos de março de 1987 a fevereiro de 1988, outubro de 1995 a setembro de 1996, junho de 2002 a julho de 2003, maio de 2008 a maio de 2009 e julho de 2017 a julho de 2018.

Nas duas primeiras edições da pesquisa, foram analisadas amostras representativas do conjunto de domicílios brasileiros situados nas principais regiões urbanas do Brasil (região metropolitana de Belém na região Norte; de Fortaleza, Recife e Salvador na região Nordeste; de Belo Horizonte, Rio de Janeiro e São Paulo na região Sudeste; de Curitiba e Porto Alegre na região Sul; e Distrito Federal e município de Goiânia, na região Centro-Oeste). Nas três edições mais recentes da pesquisa a amostra foi expandida para representar, além desses domínios, o conjunto completo dos domicílios do país. As pesquisas utilizaram um plano amostral complexo, por conglomerados em dois estágios, envolvendo sorteio dos setores censitários no primeiro estágio e domicílios no segundo. Os setores censitários são provenientes de amostra mestra do IBGE, agrupados em estratos de domicílios com alta homogeneidade geográfica e socioeconômica. Foram considerados para a construção dos estratos: a localização geográfica do setor; a situação do domicílio (urbana ou rural para as amostras com representatividade nacional); e, internamente a cada lócus geográfico, o espectro de variação socioeconômica através da renda do indivíduo responsável pelo domicílio.

As estimativas obtidas nas pesquisas com amostras nacionais representam os seguintes domínios: o país, as cinco grandes regiões (Norte, Nordeste, Sudeste, Sul e Centro-Oeste), a situação (urbana ou rural), as 26 unidades da federação e Distrito Federal, as nove regiões metropolitanas e as 26 capitais dos estados. A descrição detalhada sobre o processo de amostragem das cinco pesquisas está disponível nas publicações do IBGE ^a^ .

Para o presente estudo, foram utilizados como unidade de análise os agregados de domicílios gerados em plano amostral (estratos). Para as análises representativas de todo o país, em 2002–2003 os 48.747 domicílios resultaram em 443 estratos com o número médio de 109,4 domicílios por estrato (variando de nove a 801); em 2008–2009 os 55.970 domicílios geraram 550 estratos, com uma média de 101,7 domicílios por estrato (variando de oito a 796) e em 2017–2018 os 57.920 domicílios resultaram em 575 estratos com uma média de 86,5 domicílios por estrato (variando de 16 a 524).

Para as análises de tendência das regiões metropolitanas, foram utilizadas informações do Sistema IBGE de Recuperação Automática (Sidra) ^b^ , sobre as aquisições de alimentos e bebidas em cada uma das nove regiões metropolitanas, município de Goiânia e Distrito Federal. No Sidra encontram-se disponibilizadas informações de agregados de domicílios correspondentes a 10 classes de renda familiar, totalizando 110 estratos por pesquisa.

As informações utilizadas no presente estudo referem-se às aquisições de alimentos para consumo domiciliar feitas durante sete dias consecutivos, registradas pelos moradores do domicílio ou por um entrevistador do IBGE em uma caderneta de despesas coletiva (em medida caseira ou na própria unidade de aquisição) e convertidos em quilogramas ou litros pelo IBGE. Os alimentos consumidos fora dos domicílios não foram registrados com um nível de detalhamento satisfatório e não foram incluídos nesse estudo.

A coleta de cada pesquisa foi distribuída nos quatro trimestres do ano, incorporando a variedade sazonal, à qual as despesas estão sujeitas. As quantidades das aquisições referentes à pesquisa de 1987–1988 foram definidas indiretamente pela relação entre gastos e itens relatados, devido à ausência de dados coletados no período.

Fatores de correção foram aplicados para excluir a fração não comestível dos alimentos ^c^ . Em seguida, a quantidade comestível dos alimentos foi convertida em calorias empregando-se a Tabela Brasileira de Composição de Alimentos ^d^ .

Todos os alimentos reportados foram categorizados segundo os quatro grupos da classificação NOVA: 1) Alimentos *in natura* ou minimamente processados; 2) Ingredientes culinários processados; 3) Alimentos processados; e 4) Alimentos ultraprocessados ^3^ .

A disponibilidade domiciliar dos grupos e subgrupos de alimentos foi expressa por meio de sua participação (%) nas calorias disponíveis para consumo. A participação dos grupos e subgrupos de alimentos foi estimada para o conjunto das famílias brasileiras, por situação do domicílio (urbana ou rural), para cada uma das cinco regiões geográficas do país e segundo quintos da distribuição de renda domiciliar *per capita* (inquéritos de 2002–2003, 2008–2009 e 2017–2018) e para aquelas residindo nas 11 principais regiões urbanas do país (inquéritos de 1987–1988, 1995–1996, 2002–2003, 2008–2009 e 2017–2018).

Modelos de regressão linear foram utilizados para avaliar a tendência e a diferença entre os anos na participação dos alimentos na dieta dos brasileiros no período estudado, tendo o ano do início da pesquisa como variável explanatória e os grupos e subgrupos de alimentos como desfecho. Considerou-se p < 0,05 para significância estatística em todas as análises.

Foram utilizados fatores de ponderação, considerando a estrutura amostral e os fatores de expansão, permitindo a extrapolação dos resultados para a população brasileira. Todas as análises foram realizadas com o pacote estatístico do software Stata (StataCorp, versão 16).

## RESULTADOS

A Tabela 1 descreve a participação relativa dos grupos e subgrupos de alimentos, segundo a classificação NOVA, na disponibilidade domiciliar de alimentos em 2002–2003, 2008–2009 e 2017–2018 no Brasil. Em 2017–2018 48,7% das calorias disponíveis para consumo nos domicílios brasileiros foram de alimentos *in natura* ou minimamente processados, 21,6% de ingredientes culinários processados, 10,4% de alimentos processados e 19,4% de alimentos ultraprocessados.

**Tabela 1:** Participação relativa de grupos e subgrupos da classificação NOVA no total de calorias determinado pela aquisição de alimentos domiciliar. Brasil – períodos 2002–2003, 2008–2009 e 2017–2018.

Grupos e subgrupos de alimentos	Participação relativa, por ano da pesquisa (%)		
	2002–2003	2008–2009	2017–2018
**Alimentos in natura ou minimamente processados**	**51,0**	**48,9** ^e^	**48,7** ^f^
Arroz	16,5	15,4	15,1 ^f^
Leite	5,4	4,8 ^e^	4,7 ^f^
Carne de aves	3,1	3,6 ^e^	4,1 ^e,f^
Feijão	5,5	4,6 ^e^	4,1 ^e,f^
Carne bovina	3,3	4,3 ^e^	4,6 ^e,f^
Frutas	2,1	2,4 ^e^	2,8 ^e,f^
Macarrão	2,4	2,4	2,4
Farinha de milho, fubá e outras	1,6	1,4	1,1 ^e,f^
Farinha de mandioca	3,6	2,7	1,6 ^e,f^
Farinha de trigo	2,6	1,9 ^e^	1,7 ^f^
Raízes e tubérculos	1,1	1,1	1,2 ^e,f^
Ovos	0,3	0,7 ^e^	0,9 ^e,f^
Verduras e legumes	0,7	0,8 ^e^	0,9 ^e,f^
Carne suína	0,7	0,6	1,0 ^e,f^
Peixes	0,5	0,5	0,4
Milho, aveia e outros cereais	0,7	0,9	1,0 ^e,f^
Vísceras	0,3	0,2	0,2
Outros ^a^	0,5	0,6 ^e^	0,7 ^f^
**Ingredientes culinários processados**	**25,5**	**23,4** ^e^	**21,6** ^e,f^
Óleo vegetal	11,7	11,0	10,7 ^f^
Açúcar	12,5	11,3 ^e^	9,4 ^e,f^
Gordura animal	0,8	0,5 ^e^	0,7 ^e^
Féculas	0,4	0,5	0,7 ^e,f^
Outros ^b^	0,1	0,1	0,1 ^e,f^
**Alimentos processados**	**9,2**	**10,4** ^e^	**10,4** ^f^
Pães	6,6	7,4 ^e^	7,0
Queijos	0,9	1,1 ^e^	1,4 ^e,f^
Carnes salgadas/secas/defumadas	0,7	0,7	0,7
Bebidas alcoólicas fermentadas	0,4	0,6 ^e^	0,7 ^e,f^
Outros ^c^	0,5	0,5	0,6 ^f^
**Alimentos ultraprocessados**	**14,3**	**17,3** ^e^	**19,4** ^e,f^
Frios e embutidos	2,0	2,4 ^e^	2,7 ^e,f^
Biscoitos doces	1,9	2,1 ^e^	2,2 ^f^
Biscoitos salgados	1,4	1,6 ^e^	1,9 ^e,f^
Margarina	1,8	1,9 ^e^	1,8
Bolos e tortas doces	0,7	1,1 ^e^	1,4 ^e,f^
Pães	0,9	1,1 ^e^	1,4 ^e,f^
Doces em geral	0,5	0,7 ^e^	0,9 ^e,f^
Bebidas adoçadas carbonatadas	1,6	1,6	1,2 ^e,f^
Chocolate	0,8	1,0 ^e^	1,2 ^f^
Massas de pizza, de lasanha ou de pastel	0,4	0,6 ^e^	0,9 ^e,f^
Refeições prontas	0,4	0,7 ^e^	0,9 ^e,f^
Bebidas adoçadas não carbonatadas	0,4	0,5	0,6 ^e,f^
Bebidas lácteas	0,4	0,5 ^e^	0,5 ^e,f^
Sorvetes	0,2	0,3 ^e^	0,4 ^e,f^
Molhos prontos	0,4	0,5 ^e^	0,7 ^e,f^
Bebidas alcoólicas destiladas	0,1	0,1	0,2 ^f^
Outros ^d^	0,4	0,5	0,5

^a^ Outros alimentos *in natura* ou minimamente processados, incluindo chás e cafés, frutos do mar, carne de outros animais, nozes e sementes, frutas e hortaliças secas ou desidratadas.

^b^ Outros ingredientes culinários incluindo sal e outros açúcares.

^c^ Outros alimentos processados, incluindo peixes e frutos do mar secos e/ou salgados, conservas de cereais, leguminosas e hortaliças, castanhas salgadas e conservas de carnes diet/light.

^d^ Outros alimentos ultraprocessados, incluindo carnes reconstituídas, tabletes e temperos prontos, condimentos a base de sal sem gordura, queijos ultraprocessados e cereais matinais.

^e^ p < 0,05 na comparação com o período anterior.

^f^ p < 0,05 para tendência linear entre 2002–2003 e 2017–2018.

Dentre os alimentos *in natura* ou minimamente processados, os grupos com maior contribuição calórica foram: arroz (15,1% das calorias totais), leite (4,7%), carne bovina (4,6%), feijão (4,1%) e carne de aves (4,1%). Ainda relevantes na alimentação brasileira, apareceram frutas (2,8%), macarrão (2,4%), farinha de trigo (1,7%), farinha de mandioca (1,6%) e raízes e tubérculos (1,2%). Dentre os ingredientes culinários processados, os subgrupos com maior contribuição calórica foram: óleo vegetal (10,7%) e açúcar (9,4%). Dentre alimentos processados, os subgrupos de maior contribuição calórica foram pão (7,0%) e queijos (1,4%). Por fim, destacam-se, dentre os alimentos ultraprocessados, frios e embutidos (2,7%), biscoitos doces (2,2%), biscoitos salgados (1,9%), margarina (1,8%), bolos e tortas doces (1,4%), pães (1,4%), bebidas adoçadas carbonatadas (1,2%) e chocolate (1,2%).

Entre 2002–2003 e 2017–2018 observou-se diminuição no percentual de participação calórica de alimentos *in natura* ou minimamente processados (em média -0,15 pontos percentuais ao ano = pp/ano) e de ingredientes culinários processados (-0,24 pp/ano), em paralelo ao aumento da participação de alimentos processados (+0,07 pp/ano) e ultraprocessados (+0,31 pp/ano). O declínio na disponibilidade relativa de alimentos *in natura* ou minimamente processados foi mais intenso no primeiro período (2002–2003 a 2008–2009) do que no segundo período (2008–2009 a 2017–2018): 0,30 pp/ano e 0,03 pp/ano respectivamente. Evolução semelhante foi observada quanto à disponibilidade relativa de ingredientes culinários processados: declínio de 0,3 pp/ano no primeiro período e de 0,2 pp/ano no segundo período. Por outro lado, a intensidade do aumento na disponibilidade relativa de alimentos processados e ultraprocessados diminuiu do primeiro para o segundo período: de 0,2 pp/ano para estabilidade, no caso dos processados, e de 0,4 pp/ano para 0,2 pp/ano, no caso dos ultraprocessados.

No grupo de alimentos *in natura* ou minimamente processados, arroz (-1,4%), leite (-0,7%), feijão (-1,4%), farinha de milho, fubá e outras (-0,5%), farinha de mandioca (-2,0%) e farinha de trigo (0,9%) acompanharam a tendência do grupo, diminuindo sua participação na disponibilidade domiciliar, enquanto carne de aves (1,0%), carne bovina (1,3%), frutas (0,7%), raízes e tubérculos (0,1%), ovos (0,6%), verduras e legumes (0,2%), carne suína (0,3%) e milho, aveia e outros cereais (0,3%) aumentaram sua disponibilidade no período. Dentre os ingredientes culinários processados, foi observada queda na participação de óleo vegetal (-1,0%) e açúcar (-3,1%), em contraponto ao aumento de féculas (+0,3%). A participação de queijos (+0,5%), bebidas alcóolicas fermentadas (+0,3%) e outros alimentos processados (+0,1%) aumentou entre 2002–2003 e 2017–2018. No grupo de alimentos ultraprocessados, observou-se aumento na participação de todos os subgrupos, com exceção de margarina, que se manteve estável, e bebidas adoçadas carbonatadas (-0,4%), cuja participação diminuiu.

A Tabela 2 descreve a disponibilidade de grupos e subgrupos de alimentos, segundo quintos do rendimento domiciliar *per capita* entre 2002–2003 e 2017–2018. Em 2017–2018 a participação no total calórico de alimentos *in natura* ou minimamente processados e de ingredientes culinários processados diminuiu com o aumento da renda: no caso de alimentos *in natura* ou minimamente processados, de 57,5% no primeiro quinto para 42,9% no último; e os ingredientes culinários processados, de 21,8% no primeiro quinto para 20,0% no último. Alimentos processados e ultraprocessados aumentaram sua participação no total calórico com o aumento da renda. Esse aumento é moderado no caso dos alimentos processados, de 8,8% no primeiro quinto para 11,8% no último, e bastante intenso no caso dos alimentos ultraprocessados, de 11,9% para 25,4%. No entanto, a tendência de variação nos subgrupos com a renda foi heterogênea. Entre os alimentos *in natura* ou minimamente processados, arroz, feijão e farinha de milho, fubá e outras diminuíram sua participação com o aumento da renda, enquanto frutas, raízes e tubérculos, legumes e verduras tiveram sua participação aumentada. Nos subgrupos dos ingredientes culinários processados, com o aumento da renda observou-se redução na participação calórica de açúcar e aumento na participação de gordura animal. Entre os alimentos processados, o aumento da renda levou ao aumento na participação calórica de queijos, bebidas alcoólicas fermentadas e outros alimentos processados e redução na participação de carnes salgadas/secas/defumadas. Com a exceção de biscoitos salgados, todos os demais alimentos ultraprocessados aumentaram sua participação no total calórico com o aumento da renda.

**Tabela 2:** Participação relativa de alimentos e grupos de alimentos segundo a NOVA no total de calorias determinado pela aquisição alimentar domiciliar, por quintos de rendimento total e variação patrimonial mensal familiar e ano da pesquisa – Brasil – períodos 2002–2003, 2008–2009 e 2017–2018.

Grupos e subgrupos de alimentos	Participação relativa, por quintos de rendimento total e variação patrimonial mensal familiar (%)														
	1º Quinto			2º Quinto			3º Quinto			4º Quinto			5º Quinto		
	2002/03	2008/09	2017/18	2002/03	2008/09	2017/18	2002/03	2008/09	2017/18	2002/03	2008/09	2017/18	2002/03	2008/09	2017/18
**Alimentos in natura ou minimamente processados**	**60,0**	**57,6**	**57,5**	**54,4**	**51,3** ^e^	**50,6** ^f^	**50,2**	**47,2** ^e^	**47,3** ^f^	**47,2**	**45,7**	**44,8** ^f^	**43,0**	**42,5**	**42,9**
Arroz	17,6	18,2	19,0 ^e^	18,9	16,5 ^e^	16,5 ^f^	18,2	15,8	15,7	15,6	14,6	13,0 ^e,f^	12,1	11,9	11,3
Leite	3,8	3,5	4,1 ^e^	5,0	4,3 ^e^	4,4 ^f^	5,1	5,7	5,2	5,9	5,3 ^e^	5,1 ^f^	7,1	5,2 ^e^	5,0 ^f^
Carne de aves	2,7	4,0 ^e^	5,7 ^e,f^	3,3	3,8 ^e^	4,3 ^e,f^	3,0	3,6	3,4	3,6	3,2	3,6	2,9	3,2	3,4
Feijão	8,5	6,5 ^e^	5,2 ^e,f^	5,8	5,4	4,8 ^e,f^	5,3	4,0 ^e^	3,8 ^f^	4,4	3,6 ^e^	3,3 ^f^	3,5	3,2	3,5
Carne bovina	2,8	4,0 ^e^	4,9 ^e,f^	3,3	4,4 ^e^	4,3 ^f^	3,2	4,6 ^e^	4,6 ^f^	4,0	4,4	4,8 ^f^	3,4	4,0 ^e^	4,2 ^f^
Frutas	1,4	1,8 ^e^	2,3 ^e,f^	1,6	2,1 ^e^	2,5 ^e,f^	1,6	2,2 ^e^	2,8 ^e,f^	2,1	2,4 ^e^	3,0 ^e,f^	3,5	3,5	3,6
Macarrão	2,4	2,5	2,5	2,5	2,5	2,6	2,4	2,2	2,3	2,4	2,4	2,2	2,5	2,5	2,5
Farinha de milho, fubá e outras	3,6	3,1	2,0 ^e,f^	1,8	1,7	1,2 ^e,f^	1,4	0,9 ^e^	0,8 ^f^	0,7	0,7	0,6	0,5	0,6	0,6
Farinha de mandioca	10,5	7,2 ^e^	4,4 ^e,f^	4,0	3,5	2,0 ^e,f^	1,5	1,0	0,6 ^f^	1,0	0,7	0,5 ^f^	0,7	0,8	0,5 ^e,f^
Farinha de trigo	1,1	0,8	0,8	2,8	1,7 ^e^	1,6 ^f^	3,9	2,2 ^e^	2,0 ^f^	3,2	3,2	2,2 ^e^	2,1	1,9	1,8
Raízes e tubérculos	0,9	0,8	0,9	1,2	1,0	1,2	1,0	1,1	1,4 ^e,f^	1,3	1,3	1,4	1,3	1,2	1,4
Ovos	0,3	0,6 ^e^	0,9 ^e,f^	0,4	0,7 ^e^	1,0 ^e,f^	0,2	0,7 ^e^	0,8 ^e,f^	0,3	0,7 ^e^	0,9 ^e,f^	0,3	0,7 ^e^	1,0 ^e,f^
Verduras e legumes	0,6	0,6	0,7 ^e,f^	0,6	0,8 ^e^	0,9 ^e,f^	0,6	0,8 ^e^	1,0 ^e,f^	0,9	0,8	1,0 ^e,f^	1,0	1,0	1,1
Carne suína	0,5	0,5	0,7 ^e,f^	1,0	0,6 ^e^	0,8 ^f^	1,0	0,8	1,3 ^e,f^	0,8	0,8	1,1 ^e,f^	0,5	0,6	0,8 ^e,f^
Peixes	1,0	1,0	1,0	0,7	0,5	0,4 ^f^	0,2	0,3	0,2	0,3	0,2	0,2	0,3	0,4	0,3
Milho, aveia e outros cereais	1,3	1,6	1,4	0,7	0,9	1,2 ^f^	1,0	0,6 ^e^	0,6	0,4	0,5	1,0 ^e,f^	0,4	0,7 ^e^	0,9 ^f^
Vísceras	0,3	0,3	0,3	0,3	0,3	0,3	0,2	0,2	0,2	0,3	0,2	0,3	0,2	0,2	0,2
Outros ^a^	0,6	0,7	0,6	0,5	0,6	0,5	0,3	0,4	0,7 ^e,f^	0,4	0,6 ^e^	0,6 ^f^	0,7	0,8	0,8
**Ingredientes culinários processados**	**25,1**	**24,8**	**21,8** ^e,f^	**27,2**	**24,4** ^e^	**22,2** ^e,f^	**28,1**	**23,6** ^e^	**22,9** ^f^	**24,7**	**24,2**	**21,0** ^e,f^	**22,2**	**20,2**	**20,0**
Óleo vegetal	9,7	9,7	9,3	12,0	10,8	10,4	12,5	11,4	11,9	12,7	12,5	11,2 ^e,f^	11,5	10,8	10,9
Açúcar	14,0	13,6	11,0 ^e,f^	13,6	12,6	10,2 ^e,f^	14,1	11,2 ^e^	9,8 ^e,f^	11,0	10,9	8,7 ^e,f^	9,7	8,4 ^e^	7,3 ^e,f^
Gordura animal	0,4	0,4	0,4	1,0	0,4 ^e^	0,7 ^e^	1,1	0,7	0,6 ^f^	0,7	0,5	0,7	0,7	0,6	1,1 ^e,f^
Féculas	0,7	1,0	1,0 ^f^	0,6	0,5	0,8 ^e,f^	0,3	0,3	0,5 ^e,f^	0,2	0,3 ^e^	0,4 ^f^	0,3	0,3	0,5 ^e,f^
Outros ^b^	0,2	0,2	0,1 ^e,f^	0,1	0,1	0,1	0,1	0,1	0,1	0,1	0,1	0,1	0,1	0,1	0,1
**Alimentos processados**	**6,4**	**7,4**	**8,8** ^f^	**7,9**	**10,8** ^e^	**10,9** ^f^	**8,6**	**11,4** ^e^	**10,0**	**11,0**	**9,7**	**10,6**	**12,2**	**12,4**	**11,8**
Pães	4,5	5,6	6,9 ^f^	5,9	8,2 ^e^	8,2 ^f^	6,4	8,6 ^e^	6,5 ^e^	8,1	6,6	6,4	8,1	7,8	6,8 ^f^
Queijos	0,3	0,4 ^e^	0,6 ^e,f^	0,6	0,8 ^e^	1,1 ^e,f^	0,7	1,1 ^e^	1,4 ^e,f^	1,1	1,3	1,8 ^e,f^	2,0	2,1	2,3
Carnes salgadas/secas/defumadas	1,1	0,8 ^e^	0,8	0,7	1,0 ^e^	0,8	0,7	0,7	0,7	0,6	0,5	0,7	0,5	0,6	0,7
Bebidas alcoólicas fermentadas	0,1	0,2	0,2	0,3	0,4	0,3	0,4	0,5 ^e^	0,8 ^e,f^	0,5	0,7 ^e^	1,0 ^e,f^	0,9	1,1	1,1
Outros ^c^	0,3	0,4 ^e^	0,4	0,4	0,4	0,5 ^f^	0,4	0,5	0,5	0,6	0,6	0,7	0,7	0,8	0,8
**Alimentos ultraprocessados**	**8,6**	**10,2** ^e^	**11,9** ^e,f^	**10,5**	**13,6** ^e^	**16,3** ^e,f^	**13,0**	**17,8** ^e^	**19,9** ^e,f^	**17,2**	**20,4** ^e^	**23,6** ^e,f^	**22,6**	**24,9** ^e^	**25,4** ^f^
Frios e embutidos	0,9	1,2 ^e^	1,9 ^e,f^	1,5	2,0 ^e^	2,3 ^e,f^	2,1	3,0 ^e^	2,9 ^f^	3,0	3,0	3,5 ^e,f^	2,5	3,1 ^e^	3,2 ^f^
Biscoitos doces	1,6	1,6	1,7	1,5	1,9 ^e^	2,2 ^f^	1,9	2,2	2,2	2,0	2,1	2,4 ^e,f^	2,5	2,6	2,4
Biscoitos salgados	2,1	2,1	2,3	1,3	1,7 ^e^	2,0 ^f^	1,2	1,3	1,6 ^e,f^	1,1	1,4	1,7 ^e,f^	1,3	1,5	1,9 ^e,f^
Margarina	1,3	1,7 ^e^	1,4 ^e^	1,7	1,8	2,1 ^e^	1,6	2,0 ^e^	1,6 ^e^	1,9	2,0	1,9	2,2	2,3	2,0
Bolos e tortas doces	0,4	0,5	0,8 ^e,f^	0,5	0,8 ^e^	1,1 ^e,f^	0,6	1,0 ^e^	1,6 ^e,f^	0,9	1,5 ^e^	1,6 ^f^	1,2	1,8 ^e^	2,0 ^e,f^
Pães	0,2	0,3	0,5 ^e,f^	0,5	0,7	0,9 ^e,f^	0,7	1,0 ^e^	1,5 ^e,f^	1,0	1,5 ^e^	1,9 ^e,f^	2,0	2,2	2,2
Doces em geral	0,1	0,2 ^e^	0,3 ^e,f^	0,4	0,5	0,7 ^e,f^	0,5	0,8 ^e^	1,0 ^e,f^	0,7	1,0 ^e^	1,2 ^e,f^	1,0	1,3 ^e^	1,4 ^f^
Bebidas adoçadas carbonatadas	0,6	0,7	0,6 ^e^	1,1	1,3	0,9 ^e,f^	1,5	1,9 ^e^	1,4 ^e,f^	2,2	2,2	1,6 ^e,f^	2,4	2,1 ^e^	1,5 ^e,f^
Chocolate	0,2	0,3 ^e^	0,4 ^e,f^	0,5	0,6 ^e^	0,8 ^e,f^	0,7	1,0 ^e^	1,3 ^e,f^	1,2	1,4	1,7 ^e,f^	1,6	1,9	1,7
Massas de pizza, de lasanha ou de pastel	0,1	0,2 ^e^	0,3 ^ef^	0,2	0,4 ^e^	0,5 ^e,f^	0,3	0,6 ^e^	0,9 ^e,f^	0,5	0,8 ^e^	1,2 ^e,f^	1,1	1,0	1,3 ^e,f^
Refeições prontas	0,1	0,2 ^e^	0,4 ^e,f^	0,2	0,5 ^e^	0,7 ^e,f^	0,4	0,7 ^e^	1,0 ^e,f^	0,5	0,9 ^e^	1,2 ^e,f^	0,9	1,2 ^e^	1,5 ^f^
Bebidas adoçadas não carbonatadas	0,1	0,2 ^e^	0,2 ^e,f^	0,2	0,3 ^e^	0,4 ^e,f^	0,3	0,4	0,6 ^e,f^	0,5	0,5	0,7 ^e,f^	1,0	0,9	0,9
Bebidas lácteas	0,1	0,2 ^e^	0,2 ^f^	0,2	0,3 ^e^	0,4 ^e,f^	0,3	0,5 ^e^	0,5 ^f^	0,5	0,5	0,7 ^e,f^	0,7	0,7	0,8
Sorvetes	0,0	0,0 ^e^	0,1 ^e,f^	0,0	0,1 ^e^	0,2 ^e,f^	0,2	0,2	0,4 ^e,f^	0,2	0,3	0,6 ^e,f^	0,5	0,7	0,8 ^f^
Molhos prontos	0,1	0,1 ^e^	0,2 ^e,f^	0,2	0,3	0,5 ^e,f^	0,3	0,6 ^e^	0,8 ^e,f^	0,5	0,7 ^e^	1,0 ^e,f^	0,9	0,8	1,0 ^e,f^
Bebidas alcoólicas destiladas	0,1	0,1	0,1	0,1	0,1	0,1	0,1	0,1	0,1	0,1	0,1	0,2 ^f^	0,2	0,2	0,2
Outros ^d^	0,5	0,5	0,4 ^e,f^	0,3	0,4	0,4	0,3	0,4 ^e^	0,4 ^e,f^	0,4	0,4	0,5 ^f^	0,7	0,6	0,6

^a^ Outros alimentos *in natura* ou minimamente processados, incluindo chás e cafés, frutos do mar, carne de outros animais, nozes e sementes, frutas e hortaliças secas ou desidratadas.

^b^ Outros ingredientes culinários incluindo sal e outros açúcares.

^c^ Outros alimentos processados, incluindo peixes e frutos do mar secos e/ou salgados, conservas de cereais, leguminosas e hortaliças, castanhas salgadas e conservas de carnes diet/light.

^d^ Outros alimentos ultraprocessados, incluindo carnes reconstituídas, tabletes e temperos prontos, condimentos a base de sal sem gordura, queijos ultraprocessados e cereais matinais.

^e^ p < 0,05 na comparação com o período anterior.

^f^ p < 0,05 para tendência linear entre 2002–2003 e 2017–2018.

Ao avaliar a evolução da disponibilidade domiciliar de alimentos segundo renda, observa-se dentro dos primeiros quatro quintos de renda uma clara tendência à diminuição da disponibilidade domiciliar de alimentos *in natura* ou minimamente processados e ingredientes culinários processados, em contraponto ao aumento de alimentos ultraprocessados entre 2002–2003 e 2017–2018. Por outro lado, no quinto superior da distribuição de renda, essa tendência é observada entre 2002–2003 e 2008–2009, mas se estabiliza no período mais recente. O segundo, terceiro e quarto quintos de renda apresentaram os maiores aumentos nos percentuais de participação de alimentos ultraprocessados.

Dentre alimentos *in natura* ou minimamente processados observa-se em todos os quintos um aumento no consumo de carne bovina, ovos e carne suína. Em contraponto, o consumo de farinha de mandioca diminuiu. Ainda dentro desse subgrupo destaca-se o aumento do consumo de frutas, verduras e legumes e diminuição do consumo de feijão do primeiro ao quarto quinto de renda. Dentre ingredientes culinários processados observa-se um aumento no consumo de féculas e diminuição do consumo de açúcar em todos os quintos de renda. Dentre alimentos ultraprocessados observa-se um aumento significativo de frios e embutidos, bolos e tortas doces, doces em geral, massas de pizza, de lasanha ou de pastel, refeições prontas, sorvetes e molhos prontos em todas as classes de renda. Também se destaca o aumento na aquisição de pães ultraprocessados, chocolate, bebidas adoçadas não carbonatadas e bebidas lácteas nos quatro primeiros quintos de renda e de biscoitos salgados entre o segundo quinto de renda e o último.

A Tabela 3 retrata o percentual de participação de grupos e subgrupos de alimentos, segundo a NOVA, nas áreas urbana e rural entre 2002–2003 e 2017–2018. Comparando áreas urbana e rural, em 2017–2018 a participação de alimentos *in natura* ou minimamente processados foi maior no meio rural do que no meio urbano (58,2% contra 47,1% das calorias totais), assim como a participação de ingredientes culinários processados (24,5% contra 21,1%). Por outro lado, tanto a participação de alimentos processados quanto a de alimentos ultraprocessados foi maior no meio urbano (11,1% e 20,6%, respectivamente) do que no meio rural (5,8% e 11,5%).

**Tabela 3:** Participação relativa de grupos e subgrupos da classificação NOVA no total de calorias determinado pela aquisição alimentar domiciliar de acordo com a situação do domicílio, por ano da pesquisa – Brasil – períodos 2002–2003, 2008–2009 e 2017–2018.

Grupos e subgrupos de alimentos	Participação relativa por situação do domicílio (%)					
	Urbana			Rural		
	2002–2003	2008–2009	2017–2018	2002–2003	2008–2009	2017–2018
**Alimentos in natura ou minimamente processados**	**49,1**	**47,2** ^e^	**47,1** ^f^	**61,9**	**58,3** ^e^	**58,2** ^f^
Arroz	16,2	14,9 ^e^	14,4 ^f^	18,1	18,1	19,9
Leite	5,5	4,9 ^e^	4,8 ^f^	4,9	4,3	4,5
Carne de aves	3,2	3,6 ^e^	3,9 ^e,f^	2,3	3,2 ^e^	4,8 ^e,f^
Feijão	5,0	4,3 ^e^	4,0 ^f^	8,2	5,9 ^e^	5,0 ^e,f^
Carne bovina	3,5	4,3 ^e^	4,7 ^e,f^	2,4	4,1 ^e^	4,0 ^f^
Frutas	2,2	2,5 ^e^	2,9 ^e,f^	1,4	1,7	2,0 ^f^
Macarrão	2,5	2,5	2,4	2,0	2,2	2,5 ^f^
Farinha de milho, fubá e outras	1,3	1,2	0,9 ^e,f^	3,6	2,7	2,3 ^f^
Farinha de mandioca	2,7	2,0	1,3 ^e,f^	8,5	6,3	3,6 ^e,f^
Farinha de trigo	2,4	1,8	1,5 ^f^	3,8	2,8	2,7
Raízes e tubérculos	1,1	1,1	1,3 ^e,f^	1,4	1,1	1,2
Ovos	0,3	0,7 ^e^	0,9 ^e,f^	0,4	0,7	1,0 ^e,f^
Verduras e legumes	0,8	0,8	1,0 ^e,f^	0,5	0,6 ^e^	0,7 ^e,f^
Carne suína	0,7	0,6	0,9 ^e,f^	1,1	0,9	1,1
Peixes	0,4	0,4	0,4	0,9	0,8	0,8
Milho, aveia e outros cereais	0,6	0,7	1,0 ^e,f^	1,5	1,8	1,4
Vísceras	0,3	0,2	0,2	0,2	0,2	0,2
Outros ^a^	0,5	0,6 ^e^	0,7 ^e,f^	0,6	0,8	0,6
**Ingredientes culinários processados**	**25,2**	**22,8** ^e^	**21,1** ^e,f^	**26,8**	**26,5**	**24,5** ^e,f^
Óleo vegetal	11,9	11,0 ^e^	10,7 ^f^	10,4	10,7	10,9
Açúcar	12,2	10,8 ^e^	9,0 ^e,f^	14,2	14,3	12,3 ^e,f^
Gordura animal	0,7	0,5 ^e^	0,7 ^e^	1,1	0,5 ^e^	0,5 ^f^
Féculas	0,4	0,4	0,6 ^e,f^	0,7	0,8	0,7
Outros ^b^	0,1	0,1	0,1	0,3	0,2	0,1 ^e,f^
**Alimentos processados**	**10,1**	**11,3** ^e^	**11,1**	**3,9**	**5,2** ^e^	**5,8** ^f^
Pães	7,4	8,2	7,5 ^e^	2,0	3,2 ^e^	3,9 ^e,f^
Queijos	1,0	1,2 ^e^	1,6 ^e,f^	0,5	0,5	0,5
Carnes salgadas/secas/defumadas	0,7	0,7	0,7	1,0	0,8	0,7 ^f^
Bebidas alcoólicas fermentadas	0,5	0,6 ^e^	0,7 ^e,f^	0,1	0,3 ^e^	0,3 ^f^
Outros ^c^	0,5	0,5	0,6 ^e,f^	0,3	0,4	0,4
**Alimentos ultraprocessados**	**15,6**	**18,7** ^e^	**20,6** ^e,f^	**7,4**	**10,0** ^e^	**11,5** ^e,f^
Frios e embutidos	2,1	2,6 ^e^	2,9 ^f^	1,0	1,6 ^e^	1,9 ^e,f^
Biscoitos doces	2,0	2,2 ^e^	2,3 ^f^	1,3	1,5	1,6
Biscoitos salgados	1,4	1,6 ^e^	1,9 ^e,f^	1,4	1,6	2,0 ^e,f^
Margarina	1,9	2,1	1,9 ^e^	0,8	1,2 ^e^	1,2 ^f^
Bolos e tortas doces	0,8	1,2 ^e^	1,6 ^e,f^	0,3	0,5 ^e^	0,7 ^e,f^
Pães	1,0	1,3 ^e^	1,5 ^e,f^	0,5	0,5	0,5
Doces em geral	0,6	0,8 ^e^	1,0 ^e,f^	0,2	0,3 ^e^	0,4 ^f^
Bebidas adoçadas carbonatadas	1,8	1,8	1,3 ^e,f^	0,6	0,8 ^e^	0,6 ^e^
Chocolate	0,9	1,1 ^e^	1,3 ^f^	0,3	0,5 ^e^	0,6 ^f^
Massas de pizza, de lasanha ou de pastel	0,5	0,7 ^e^	0,9 ^e,f^	0,1	0,2 ^e^	0,4 ^e,f^
Refeições prontas	0,5	0,8 ^e^	1,0 ^e,f^	0,1	0,3 ^e^	0,4 ^e,f^
Bebidas adoçadas não carbonatadas	0,5	0,5	0,6 ^e,f^	0,1	0,2 ^e^	0,3 ^f^
Bebidas lácteas	0,4	0,5 ^e^	0,6 ^e,f^	0,1	0,2 ^e^	0,2 ^e,f^
Sorvetes	0,2	0,3 ^e^	0,5 ^e,f^	0,0	0,1 ^e^	0,1 ^e,f^
Molhos prontos	0,4	0,5 ^e^	0,8 ^e,f^	0,1	0,2 ^e^	0,3 ^e,f^
Bebidas alcoólicas destiladas	0,1	0,1	0,2	0,1	0,1	0,1
Outros ^d^	0,4	0,5	0,5	0,3	0,3	0,3

^a^ Outros alimentos *in natura* ou minimamente processados, incluindo chás e cafés, frutos do mar, carne de outros animais, nozes e sementes, frutas e hortaliças secas ou desidratadas.

^b^ Outros ingredientes culinários incluindo sal e outros açúcares.

^c^ Outros alimentos processados, incluindo peixes e frutos do mar secos e/ou salgados, conservas de cereais, leguminosas e hortaliças, castanhas salgadas e conservas de carnes diet/light.

^d^ Outros alimentos ultraprocessados, incluindo carnes reconstituídas, tabletes e temperos prontos, condimentos a base de sal sem gordura, queijos ultraprocessados e cereais matinais.

^e^ p < 0,05 na comparação com o período anterior.

^f^ p < 0,05 para tendência linear entre 2002–2003 e 2017–2018.

Tendências de consumo na área urbana e rural seguem tendências nacionais, observando-se uma queda no consumo de alimentos *in natura* ou minimamente processados e ingredientes culinários processados entre 2002–2003 e 2017–2018, em detrimento do aumento no consumo de alimentos ultraprocessados. Na área rural também foi observada tendência positiva e significativa no percentual de participação de alimentos processados.

A Tabela 4 descreve a disponibilidade de grupos e subgrupos de alimentos, segundo as grandes regiões do país. Em 2017–2018 a participação dos alimentos *in natura* ou minimamente processados no total calórico da disponibilidade alimentar foi maior no Norte e Nordeste (58,3% e 54,2%, respectivamente) e menor no Sudeste, Sul e Centro-Oeste (44,3%, 46,2% e 50,5%). A participação de ingredientes culinários processados e de alimentos processados no total calórico apresentou menores variações, ficando, respectivamente, entre 19,9%–23,9% e 8,3%–11,6% em todas as regiões. A participação de alimentos ultraprocessados no total calórico da disponibilidade alimentar foi maior no Sul, Sudeste e Centro-Oeste (23,5%, 22,5% e 17,3%) e menor no Norte e Nordeste (11,9%, e 14,3%).

**Tabela 4:** Participação relativa de grupos e subgrupos da classificação NOVA no total de calorias determinado pela aquisição alimentar domiciliar de acordo com as grandes regiões do Brasil, por ano da pesquisa – Brasil – períodos 2002–2003, 2008–2009 e 2017–2018.

Grupos e subgrupos de alimentos	Participação relativa de acordo com a região do domicílio (%)														
	Norte			Nordeste			Sudeste			Sul			Centro-Oeste		
	2002/03	2008/09	2017/18	2002/03	2008/09	2017/18	2002/03	2008/09	2017/18	2002/03	2008/09	2017/18	2002/03	2008/09	2017/18
**Alimentos in natura ou minimamente processados**	**62,4**	**58,4** ^e^	**58,3** ^f^	**56,1**	**53,8**	**54,2**	**46,2**	**44,4** ^e^	**44,3** ^f^	**51,2**	**47,7** ^e^	**46,2** ^f^	**52,5**	**52,0**	**50,5**
Arroz	17,1	15,5	18,3	16	15,1	15,8	16,9	15,5	14,9 ^f^	12,5	11,9	11,1	24,7	22,8	19,3 ^e,f^
Leite	3,8	3,4	3,8	4,2	3,9 ^e^	4,3 ^e^	5,9	5,3 ^e^	5,0 ^f^	6,2	5,8	5,4 ^f^	5,8	4,4 ^e^	4,4 ^f^
Carne de aves	3,8	4,8 ^e^	6,1 ^e,f^	2,9	4,3 ^e^	5,4 ^e,f^	3,2	3,2	3,1	3,1	3,2	3,6 ^e,f^	2,8	2,9	4,0 ^e,f^
Feijão	4,5	4,4	3,7 ^e,f^	8,3	6,3 ^e^	5,5 ^e,f^	4,8	4,1	4,0 ^f^	3,9	2,9 ^e^	2,8 ^f^	4,8	4,4	3,7 ^e,f^
Carne bovina	4,4	5,4 ^e^	6,2 ^e,f^	3,0	4,1 ^e^	4,5 ^f^	3,0	3,6 ^e^	3,9 ^f^	4,2	5,4 ^e^	5,1 ^f^	3,9	5,6 ^e^	6,1 ^f^
Frutas	2,8	2,5	2,8	1,6	2,3	2,6 ^e,f^	2,1	2,5	2,9 ^e,f^	2,5	2,5	2,9 ^e^	1,5	2,1 ^e^	2,8 ^e,f^
Macarrão	1,7	2,1	1,8	2,7	2,6	2,8	2,4	2,4	2,3	2,5	2,6	2,6	1,8	1,8	1,6 ^e,f^
Farinha de milho, fubá e outras	0,8	1,0	0,9	3,5	3,0 ^e^	2,1 ^e,f^	1,0	0,9	0,7 ^e,f^	1,3	0,9 ^e^	0,8 ^f^	0,6	0,7	0,5
Farinha de mandioca	15,4	11,5	7,6 ^e,f^	8,1	5,5 ^e^	3,0 ^e,f^	0,8	0,7	0,5 ^e,f^	0,5	0,4	0,3 ^e,f^	0,8	0,8	0,5 ^e,f^
Farinha de trigo	1,1	1,1	1,1	0,9	0,6	0,7	1,9	1,5	1,3 ^f^	8,3	5,9 ^e^	4,9 ^f^	2,1	1,8	1,4 ^f^
Raízes e tubérculos	0,8	0,7	0,5 ^f^	0,9	0,9 ^e^	1,2 ^e,f^	1,1	1,1	1,2 ^e^	1,7	1,7	1,7	0,8	1,0 ^e^	1,3 ^e,f^
Ovos	0,5	0,6 ^e^	0,8 ^f^	0,4	0,7	1,1 ^e,f^	0,0	0,7 ^e^	0,9 ^e,f^	0,9	0,8	1,0 ^e^	0,4	0,5 ^e^	0,9 ^e,f^
Verduras e legumes	0,5	0,6	0,6	0,7	0,7	0,9 ^e,f^	0,8	0,9	1,0 ^e,f^	0,7	0,8 ^e^	0,9 ^e,f^	0,7	0,8 ^e^	1,0 ^e,f^
Carne suína	0,6	0,4	0,4	0,4	0,3	0,6 ^e,f^	0,7	0,7	1,1 ^e,f^	1,5	1,1 ^e^	1,3	0,8	0,6	1,3 ^e,f^
Peixes	2,8	2,2	1,8 ^f^	0,7	0,7 ^e^	0,6	0,3	0,3	0,2 ^e^	0,2	0,2	0,2	0,2	0,2	0,3 ^f^
Milho, aveia e outros cereais	0,5	0,8 ^e^	0,8 ^f^	1,0	1,5	2,0 ^e,f^	0,7	0,5	0,6	0,5	0,7	0,8	0,4	1,0 ^e^	0,9
Vísceras	0,4	0,3	0,3	0,3	0,3 ^e^	0,3	0,2	0,2	0,2	0,2	0,2	0,3	0,2	0,2	0,2
Outros ^a^	0,9	1,0	0,7	0,6	0,8 ^e^	0,8 ^f^	0,4	0,4	0,6 ^e,f^	0,5	0,8 ^e^	0,6	0,3	0,5 ^e^	0,6 ^f^
**Ingredientes culinários processados**	**22,2**	**22,4**	**21,2**	**23,8**	**22,1**	**19,9** ^e,f^	**26,6**	**24,2** ^e^	**22,3** ^e,f^	**24,2**	**22,4** ^e^	**21,4** ^f^	**30,4**	**26,4** ^e^	**23,9** ^e,f^
Óleo vegetal	10,1	10,0	9,9	8,7	8,4 ^e^	7,8 ^e,f^	13,0	12,1	12,0	11,3	11,4	11,5	16,2	13,9 ^e^	13,3 ^f^
Açúcar	10,5	11,0	9,5 ^e,f^	13,6	12,2	10,3 ^e,f^	12,6	11,4	9,3 ^e,f^	10,9	9,7	8,7 ^f^	13,1	11,5 ^e^	8,8 ^e,f^
Gordura animal	0,6	0,5	0,5	0,4	0,5	0,6 ^e,f^	0,7	0,5 ^e^	0,7 ^e^	1,6	0,8 ^e^	0,8 ^f^	0,7	0,5	1,0
Féculas	0,9	0,9	1,2	0,8	0,9	1,1 ^e,f^	0,2	0,3	0,4 ^f^	0,2	0,2	0,4 ^e,f^	0,4	0,5	0,7 ^e,f^
Outros ^b^	0,1	0,1	0,0	0,2	0,2 ^e^	0,1 ^e,f^	0,0	0,1	0,0	0,2	0,1	0,1	0,1	0,1	0,2
**Alimentos processados**	**7,2**	**7,9**	**8,6**	**9,0**	**10,9** ^e^	**11,6** ^f^	**10,6**	**11,5**	**10,9**	**7,4**	**8,6** ^e^	**8,9**	**6,8**	**7,6**	**8,3** ^f^
Pães	5,9	6,3	6,7	6,6	8,2 ^e^	8,9 ^f^	7,6	8,2	6,9 ^e^	4,6	5,3	4,9 ^f^	4,8	5,3	5,0
Queijos	0,3	0,4	0,5	0,5	0,9	1,0 ^f^	1,2	1,3	1,8 ^e,f^	1,2	1,5	1,7 ^f^	0,7	0,8	1,3 ^e,f^
Carnes salgadas/secas/defumadas	0,6	0,6	0,7	1,2	1,1	0,9 ^f^	0,7	0,7	0,7	0,3	0,4	0,4	0,4	0,4	0,6 ^e,f^
Bebidas alcoólicas fermentadas	0,2	0,3	0,3	0,2	0,3	0,3	0,5	0,7 ^e^	0,8 ^f^	0,6	0,8	1,1 ^e,f^	0,5	0,6	0,8 ^e,f^
Outros ^c^	0,3	0,4 ^e^	0,4	0,4	0,5 ^e^	0,5	0,6	0,5	0,6	0,6	0,7	0,8 ^f^	0,4	0,5	0,5
**Alimentos ultraprocessados**	**8,2**	**11,3** ^e^	**11,9** ^f^	**11,2**	**13,2** ^e^	**14,3** ^f^	**16,6**	**19,8** ^e^	**22,5** ^e,f^	**17,2**	**21,4** ^e^	**23,5** ^e,f^	**10,3**	**13,9** ^e^	**17,3** ^e,f^
Frios e embutidos	1,0	1,4 ^e^	1,8 ^f^	1,1	1,4	1,9 ^e,f^	2,6	3,1 ^e^	3,3 ^f^	2,4	3,0 ^e^	3,3 ^e,f^	1,3	1,9 ^e^	2,3 ^e,f^
Biscoitos doces	0,8	1,1 ^e^	1,1	1,9	2,0	2,2	2,1	2,3	2,3	2,1	2,2	2,3	1,4	1,6	1,9 ^f^
Biscoitos salgados	1,4	1,7 ^e^	1,6	2,4	2,3	2,5	1,0	1,3 ^e^	1,8 ^e,f^	1,1	1,3	1,4	0,7	1,1 ^e^	1,5 ^e,f^
Margarina	1,4	1,6	1,5	2,0	2,1 ^e^	1,9	1,7	2,0	1,9	1,8	1,9	1,7	1,3	1,5	1,3 ^e^
Bolos e tortas doces	0,3	0,6 ^e^	0,6 ^f^	0,5	0,8 ^e^	1,1 ^e,f^	0,8	1,2 ^e^	1,7 ^e,f^	1,0	1,8 ^e^	1,8 ^f^	0,5	0,7 ^e^	1,4 ^e,f^
Pães	0,5	0,8 ^e^	1,0 ^e,f^	0,3	0,5 ^e^	0,5 ^f^	1,0	1,3 ^e^	1,7 ^e,f^	1,9	2,0	2,2	0,6	1,0 ^e^	1,4 ^e,f^
Doces em geral	0,2	0,4 ^e^	0,6 ^e,f^	0,2	0,4	0,4 ^f^	0,7	0,9 ^e^	1,1 ^e,f^	0,7	1,0 ^e^	1,3 ^e,f^	0,4	0,7 ^e^	0,9 ^e,f^
Bebidas adoçadas carbonatadas	0,9	1,2 ^e^	0,7 ^e,f^	0,8	0,9 ^e^	0,7 ^e,f^	2,0	2,0	1,4 ^e,f^	1,9	2,0	1,7 ^e,f^	1,5	1,6	1,2 ^e,f^
Chocolate	0,3	0,6 ^e^	0,5 ^f^	0,3	0,4 ^e^	0,5 ^f^	1,1	1,3	1,5 ^f^	1,2	1,6 ^e^	1,8 ^f^	0,6	0,9 ^e^	1,0 ^e,f^
Massas de pizza, de lasanha ou de pastel	0,1	0,3 ^e^	0,4 ^e,f^	0,2	0,3 ^e^	0,4 ^e,f^	0,6	0,8 ^e^	1,1 ^e,f^	0,6	0,9 ^e^	1,1 ^e,f^	0,3	0,5	0,9 ^e,f^
Refeições prontas	0,2	0,3 ^e^	0,5 ^e,f^	0,2	0,3 ^e^	0,4 ^e,f^	0,6	0,9 ^e^	1,2 ^e,f^	0,5	1,0 ^e^	1,2 ^e,f^	0,3	0,6 ^e^	1,0 ^e,f^
Bebidas adoçadas não carbonatadas	0,2	0,4 ^e^	0,2 ^e^	0,1	0,2 ^e^	0,2 ^f^	0,6	0,6	0,7 ^e^	0,4	0,6 ^e^	0,8 ^e,f^	0,3	0,5 ^e^	0,6 ^e,f^
Bebidas lácteas	0,1	0,3 ^e^	0,3 ^f^	0,2	0,4 ^e^	0,4 ^f^	0,5	0,5	0,6 ^e,f^	0,4	0,6 ^e^	0,7 ^e,f^	0,2	0,4 ^e^	0,4 ^f^
Sorvetes	0,0	0,1 ^e^	0,2 ^e,f^	0,1	0,1	0,2 ^e,f^	0,3	0,4	0,6 ^e,f^	0,2	0,4 ^e^	0,6 ^e,f^	0,2	0,2	0,5 ^e,f^
Molhos prontos	0,2	0,2 ^e^	0,4 ^e,f^	0,2	0,2	0,4 ^e,f^	0,5	0,6	0,9 ^e,f^	0,6	0,8 ^e^	1,1 ^e,f^	0,3	0,4 ^e^	0,6 ^e,f^
Bebidas alcoólicas destiladas	0,0	0,1 ^e^	0,2	0,1	0,1	0,1	0,1	0,1	0,2	0,2	0,1	0,2 ^e^	0,1	0,1 ^e^	0,1
Outros ^d^	0,3	0,3	0,3	0,6	0,7	0,5 ^e,f^	0,4	0,4	0,5 ^e^	0,2	0,3 ^e^	0,4 ^e,f^	0,2	0,3	0,4 ^f^

^a^ Outros alimentos *in natura* ou minimamente processados, incluindo chás e cafés, frutos do mar, carne de outros animais, nozes e sementes, frutas e hortaliças secas ou desidratadas.

^b^ Outros ingredientes culinários incluindo sal e outros açúcares.

^c^ Outros alimentos processados, incluindo peixes e frutos do mar secos e/ou salgados, conservas de cereais, leguminosas e hortaliças, castanhas salgadas e conservas de carnes diet/light.

^d^ Outros alimentos ultraprocessados, incluindo carnes reconstituídas, tabletes e temperos prontos, condimentos a base de sal sem gordura, queijos ultraprocessados e cereais matinais.

^e^ p < 0,05 na comparação com o período anterior.

^f^ p < 0,05 para tendência linear entre 2003 e 2018.

Ao avaliar a tendência de aquisição de alimentos entre 2002–2003 e 2017–2018, segundo grandes regiões, foi observada queda significativa na aquisição de alimentos *in natura* ou minimamente processados no Norte, Sudeste e Sul. Dentre alimentos desse grupo houve queda no consumo de feijão e farinha de mandioca em todas as regiões, em contraponto ao aumento de carne bovina. O consumo de ingredientes culinários processados decresceu em todas as regiões, com exceção do Norte, cuja participação se manteve relativamente estável entre 2002–2003 e 2017–2018. Em todas as regiões observou-se declínio significativo na participação do açúcar nas compras. O consumo de alimentos processados se manteve estável no Norte, Sudeste e Sul, e aumentou nas regiões Nordeste e Centro-Oeste. O aumento da aquisição de alimentos ultraprocessados foi observado em todas as regiões. Tendências na aquisição de bebidas adoçadas carbonatadas no período avaliado se mostraram bastante semelhantes nas cinco regiões, notando crescimento no seu consumo entre 2002–2003 e 2008–2009 seguido de queda entre 2008–2009 e 2017–2018.

A Tabela 5 descreve a evolução da disponibilidade domiciliar de grupos e subgrupos de alimentos com base nas POFs das regiões metropolitanas do país, realizadas em 1987–1988, 1995–1996, 2002–2003, 2008–2009 e 2017–2018. Nesse longo período, observa-se declínio da participação de alimentos *in natura* ou minimamente processados e ingredientes culinários processados, e aumento no percentual relativo dos alimentos processados e ultraprocessados. Em 1987–1988 a participação nas calorias da soma do grupo de alimentos *in natura* ou minimamente processados e ingredientes culinários processados perfazia cerca de 80% das calorias consumidas, enquanto os alimentos ultraprocessados contribuíam com apenas 10% da participação calórica. O período de maior crescimento na participação de alimentos ultraprocessados e consequente diminuição dos alimentos *in natura* ou minimamente processados e ingredientes culinários ocorreu entre 1995 e 2003, quando a taxa de crescimento anual da participação de alimentos ultraprocessados foi de 0,8%, enquanto nos demais períodos a taxa de crescimento observada ficou perto de 0,2 a 0,3 pontos percentuais ao ano. Após 30 anos, em 2017, a soma da participação dos alimentos *in natura* ou minimamente processados com os ingredientes culinários passaram a representar 64% das calorias da alimentação, enquanto os alimentos ultraprocessados atingiram cerca de 24% delas.

**Tabela 5:** Participação relativa de alimentos e grupos de alimentos da classificação NOVA no total de calorias determinado pela aquisição alimentar domiciliar nas regiões metropolitanas – períodos 1987–1988, 1995–1996, 2002–2003, 2008–2009 e 2017–2018.

Grupos e subgrupos de alimentos	Participação relativa, por ano (%)				
	1987–1988	1995–1996	2002–2003	2008–2009	2017–2018
**Alimentos in natura ou minimamente processados**	**51,5**	**50,9**	**45,8**	**44,7**	**44,9** ^f^
Arroz	15,8	15,6	13,9	14,2	12,2 ^f^
Leite	6,5	6,8	6,0	5,2	5,2 ^f^
Carne de aves	3,6	4,9	4,2	3,7	4,0
Feijão	5,3	5,0	5,0	4,5	4,2 ^f^
Carne bovina	3,2	4,0	3,4	3,5	5,0 ^f^
Frutas	2,7	2,5	2,4	2,6	3,2 ^f^
Macarrão	2,2	2,2	2,7	2,7	2,9 ^f^
Farinha de milho, fubá e outras	1,2	1,1	0,9	1,0	0,8 ^f^
Farinha de mandioca	2,7	2,2	2,0	1,5	1,2 ^f^
Farinha de trigo	2,2	1,8	1,6	1,4	1,3 ^f^
Raízes e tubérculos	1,4	1,1	1,2	1,1	1,3
Ovos	1,5	1,1	0,2	0,9	1,0 ^f^
Verduras e legumes	1,0	0,9	0,8	0,8	1,0
Carne suína	0,8	0,5	0,4	0,4	0,7
Peixes	0,4	0,4	0,4	0,4	0,3
Milho, aveia e outros cereais	0,1	0,2	0,3	0,2	0,3 ^f^
Vísceras	0,8	0,4	0,4	0,3	0,3 ^f^
Outros ^a^	0,1	0,1	0,2	0,2	0,2 ^f^
**Ingredientes culinários processados**	**27,3**	**25,3**	**23,1**	**21,4**	**19,4** ^f^
Óleo vegetal	12,3	11,0	11,1	9,8	9,9 ^f^
Açúcar	13,3	13,0	10,6	10,0	7,7 ^f^
Gordura animal	1,1	0,8	1,0	1,2	1,1
Féculas	0,6	0,4	0,3	0,2	0,6
Outros ^b^	0,1	0,0	0,1	0,1	0,1 ^f^
**Alimentos processados**	**11,1**	**11,9**	**12,9**	**13,2**	**12,1** ^f^
Pães	9,2	9,7	10,0	10,3	8,1
Queijos	0,9	1,1	1,3	1,4	2,0 ^f^
Carnes salgadas/secas/defumadas	0,4	0,4	0,8	0,7	0,8 ^f^
Bebidas alcoólicas fermentadas	0,3	0,5	0,5	0,6	0,8 ^f^
Outros ^c^	0,3	0,2	0,2	0,2	0,4 ^f^
**Alimentos ultraprocessados**	**10,2**	**12,0**	**18,2**	**20,7**	**23,7** ^f^
Frios e embutidos	0,7	1,7	2,5	2,8	2,9 ^f^
Biscoitos doces	1,1	1,6	2,2	2,2	2,4 ^f^
Biscoitos salgados	0,9	1,2	1,3	1,5	1,7 ^f^
Margarina	2,0	1,5	2,3	2,4	2,2 ^f^
Bolos e tortas doces	0,6	0,6	0,4	1,1	1,7 ^f^
Pães	0,6	0,4	1,0	1,2	2,0 ^f^
Doces em geral	1,2	0,7	0,9	1,2	1,6 ^f^
Bebidas adoçadas carbonatadas	0,8	1,2	2,0	2,0	1,4 ^f^
Chocolate	0,4	0,4	0,9	0,9	1,2 ^f^
Massas de pizza, de lasanha ou de pastel	0,2	0,4	0,5	0,5	0,8 ^f^
Refeições prontas	0,4	0,8	1,0	1,3	1,8 ^f^
Bebidas adoçadas não carbonatadas	0,2	0,4	1,3	1,6	0,9 ^f^
Bebidas lácteas	0,1	0,1	0,5	0,5	0,6 ^f^
Sorvetes	0,1	0,2	0,3	0,3	0,6 ^f^
Molhos prontos	0,2	0,2	0,4	0,4	0,5 ^f^
Bebidas alcoólicas destiladas	0,2	0,1	0,1	0,1	0,2
Outros ^d^	0,4	0,4	0,5	0,6	1,2 ^f^

^a^ Outros alimentos *in natura* ou minimamente processados, incluindo chás e cafés, frutos do mar, carne de outros animais, nozes e sementes, frutas e hortaliças secas ou desidratadas.

^b^ Outros ingredientes culinários incluindo sal e outros açúcares.

^c^ Outros alimentos processados, incluindo peixes e frutos do mar secos e/ou salgados, conservas de cereais, leguminosas e hortaliças, castanhas salgadas e conservas de carnes diet/light.

^d^ Outros alimentos ultraprocessados, incluindo carnes reconstituídas, tabletes e temperos prontos, condimentos a base de sal sem gordura, queijos ultraprocessados e cereais matinais.

^f^ p < 0,05 para tendência linear entre 1987–1988 e 2017–2018.

## DISCUSSÃO

Os resultados deste estudo mostram que ainda predominam na dieta da população brasileira alimentos *in natura* ou minimamente processados e ingredientes culinários, base das preparações culinárias tradicionalmente consumidas no país. Nas regiões Norte, Centro-Oeste e Nordeste, no meio rural e entre famílias com menor renda, a participação de alimentos *in natura* ou minimamente processados e de ingredientes culinários contribuem com mais de 50% das calorias adquiridas diariamente. Nas regiões Sul e Sudeste, nas regiões metropolitanas e meio urbano e entre famílias com maior renda, embora alimentos *in natura* ou minimamente processados e ingredientes culinários processados ainda predominem, alimentos ultraprocessados já representam mais de um quinto das calorias adquiridas pelos domicílios.

Nossos resultados ainda reforçam a tendência de aumento da participação de alimentos ultraprocessados em detrimento do consumo das preparações culinárias ^11^ . Mas indicam, também, uma desaceleração desse aumento que foi de 0,4 pp/ano entre 2002 e 2009 e passou para 0,2 pp/ano entre 2008 e 2018. Tendências semelhantes foram encontradas em domicílios urbanos e rurais, em todas as regiões e níveis de renda no país. Nas regiões metropolitanas, para o período de cerca de 30 anos, a participação relativa de alimentos ultraprocessados na dieta aumentou de 10,2% para 23,7% do total calórico, representando um aumento de 13,5% (mais de 130% de aumento), sendo o intervalo de 1995 a 2003 o que apresentou a maior taxa de crescimento, 0,8% ao ano. Acompanhando a tendência do país, também se observa a diminuição da velocidade de crescimento desse grupo de alimentos, no período mais recente.

De maneira oposta aos demais alimentos *in natura* ou minimamente processados, observou-se entre 2002–2003 e 2017–2019 um aumento na disponibilidade de frutas e de carne bovina na dieta da população. O consumo de frutas é considerado um marcador de alimentação saudável, por estar associado à proteção contra o ganho de peso excessivo e o desenvolvimento de várias doenças crônicas não transmissíveis ^12^ . Ainda que esse aumento seja visto como um aspecto positivo no padrão de alimentação, o consumo de frutas pela população brasileira (de aproximadamente 54,9 g/dia – dados não tabulados) permanece, ainda, muito abaixo se considerarmos a recomendação da Organização Mundial da Saúde (de 400g diárias) ^12^ . Com relação à carne bovina, apesar de pertencer ao grupo de alimentos *in natura* ou minimamente processados, seu consumo excessivo está associado ao desenvolvimento de doenças, como certos tipos de cânceres, doenças cardiovasculares e outras ^15^ , além de resultar em um alto impacto ambiental ^19^ . Vale destacar que o aumento na participação de carne bovina na dieta da população é observado em todas as faixas de renda, tendo a maior intensidade na menor faixa de renda (aquela com menor consumo no início do período estudado).

Os subgrupos de alimentos ultraprocessados apresentaram, de maneira geral, tendência semelhante, aumentando entre 1987–1988 e 2017–2018 nas regiões metropolitanas e entre 2002–2003 e 2017–2018 em todo território nacional. Destaca-se o aumento da aquisição de frios e embutidos, bolos e tortas doces, biscoitos salgados, pães ultraprocessados e refeições prontas. Entre os subgrupos de alimentos ultraprocessados a exceção foi o subgrupo de bebidas adoçadas carbonatadas que apresentou aumento nos primeiros períodos (1987–1988 a 2002–2003), observada apenas para as regiões metropolitanas pela maior série temporal em análise, seguido de estabilidade e queda, tanto para as regiões metropolitanas quanto para o país, sendo a queda mais acentuada entre domicílios de maior poder econômico.

A diminuição no consumo de bebidas adoçadas carbonatadas encontrada neste estudo também vem sendo observada nas capitais pelo Sistema de Monitoramento de Fatores de Risco e Proteção para Doenças Crônicas ^20^ resultado de uma provável conscientização dos seus malefícios. Os riscos à saúde associados ao consumo de bebidas açucaradas, em especial refrigerantes, são amplamente apontados pela literatura ^21^ , cuja recomendação é para diminuição do consumo de refrigerante e outras bebidas açucaradas, segundo a Organização Pan-americana de Saúde ^25^ e a Organização Mundial da Saúde ^26 , 27^ .

O aumento no consumo dos demais subgrupos de alimentos ultraprocessados, ainda que tenhamos observado a queda na velocidade de crescimento, permanece preocupante. Os resultados mostram que esse consumo é maior nos domicílios de maior renda, nas regiões mais desenvolvidas, Sul e Sudeste (22,5% e 23,5%), na área urbana (28,6%), e nas regiões metropolitanas (23,7%). Há na literatura científica um robusto corpo de evidências que associam o consumo desse grupo de alimentos com a piora na qualidade da dieta, com maior percentual de açúcar livre, gordura total e saturada, menor concentração de fibras e proteínas e menor teor de vários minerais e vitaminas ^28^ . Para além da qualidade da dieta, evidências associam a maior participação desse grupo de alimentos com o maior risco de ganho de peso e obesidade, diabetes, doenças cardiovasculares, entre outras ^2 , 5^ .

Apesar da tendência de aumento, a participação de alimentos ultraprocessados no Brasil ainda é menor, quando comparada a países de renda *per capita* maior, como EUA, Canadá, Reino Unido e Austrália ^29^ , e em outros países de renda média, como Chile e México ^33 , 34^ . O Brasil possui uma cultura alimentar rica e diversa, e cada região possui preparações com alimentos regionais tradicionais ^35^ , o que poderia explicar, pelo menos em parte, a predominância das preparações culinárias no padrão alimentar brasileiro. Outra possível explicação para isso, são os preços relativos desses alimentos no país. No Brasil, o padrão alimentar baseado em alimentos ultraprocessados ainda é mais caro que o padrão baseado em alimentos *in natura* ou minimamente processados ^36^ . Entretanto análises de projeção apontam tendência de inversão desse incentivo, em função da constante redução relativa nos preços de alimentos ultraprocessados e aumento no preço de alimentos *in natura* , observada desde o início dos anos 2000 ^37^ . É natural acreditar que essa tendência exerça influência sobre o aumento na participação dos alimentos ultraprocessados observada no período. Deve-se ter em mente ainda que, paralelamente ao cenário de mudança nos preços, a partir de 2014, o país vivenciou significativa crise econômica, com período de pressão inflacionária e redução da renda de parcela expressiva da população ^38 , 39^ .

Ações educativas também podem ter influenciado significativamente nesse processo, especialmente a partir da publicação do *Guia Alimentar para a População Brasileira* , em 2014 ^4^ . O Guia foi pioneiro ao considerar o grau de processamento dos alimentos em suas recomendações e orientar diretamente que se evite o consumo de alimentos ultraprocessados. O documento iniciou um movimento de conscientização de profissionais da saúde e da população sobre os malefícios do consumo de alimentos ultraprocessados e, desde então, tem servido como base para a criação de políticas públicas, visando a diminuição do consumo desses alimentos. Um exemplo é a publicação da resolução n. 6 de 8 de maio de 2020, que trouxe um maior alinhamento do Programa Nacional de Alimentação Escolar com o Guia Alimentar, limitando a aquisição de alimentos processados e ultraprocessados com recursos do programa a 20%, no máximo, e proibindo a oferta de alimentos ultraprocessados para crianças de até três anos de idade ^40^ .

Apesar desses avanços e da desaceleração do crescimento da participação dos alimentos ultraprocessados na dieta dos brasileiros, ações e políticas implementadas até o momento não foram suficientes para conter o aumento geral desse grupo, em especial entre as faixas de renda mais baixas. Promover a alimentação adequada e saudável implica no engajamento e na articulação de diferentes setores e atores que precisam avançar em medidas que promovam ambientes saudáveis em espaços institucionais, como a regulamentação da venda de alimentos ultraprocessados nas cantinas escolares; medidas de proteção, como a regulamentação da publicidade de alimentos ultraprocessados, principalmente direcionados ao público infantil; e políticas de preço e tributação. Países como, México, França, Reino Unido e Hungria, por exemplo, adotaram políticas de taxação de bebidas açucaradas ^41^ e os primeiros resultados no México, por exemplo, mostraram uma redução de 6% na compra dessas bebidas ^45^ . Políticas semelhantes poderiam ser incorporadas e expandidas para os demais alimentos ultraprocessados no Brasil.

É importante mencionar que mudanças no contexto político nos últimos anos colocam em risco avanços até o momento conquistados. Em nota técnica publicada em setembro de 2020, o Ministério da Agricultura, Pecuária e Abastecimento reivindicou a revisão do *Guia Alimentar da População Brasileira* sob o falso preceito de que não existiam evidências científicas que embasassem as recomendações e diretrizes do Guia ^46^ . A nota publicada ocasionou repúdio de organizações e instituições acadêmicas do Brasil e de outros países que se manifestaram em defesa do Guia Alimentar Brasileiro. Um número crescente de estudos científicos evidencia a associação entre alimentos ultraprocessados e um declínio na qualidade da dieta e desfechos em saúde ^2 , 5^ . Outros países, como a França, Canadá e Uruguai, têm adotado metas de diminuição do consumo de alimentos ultraprocessados em suas políticas públicas ^47^ . Isso expõe a fragilidade de políticas de alimentação e nutrição no Brasil, decorrentes de pressões econômicas.

## CONCLUSÕES

O presente estudo destaca-se por avaliar a disponibilidade de alimentos nos domicílios brasileiros, considerando os grupos e subgrupos da classificação NOVA, que é reconhecida internacionalmente e tem sido utilizada nas recomendações de guias alimentares. Embora o estudo avalie a alimentação dos brasileiros baseando-se somente na disponibilidade de alimentos para consumo dentro do domicílio e não o seu efetivo consumo pelos indivíduos, esses dados são úteis para o acompanhamento do padrão de alimentação da população brasileira, sobretudo quando os indicadores utilizados focalizam na participação relativa e não nas quantidades absolutas dos alimentos e quando a alimentação dentro de casa representa em torno de 70% das calorias ingeridas pela população ^50^ . Mesmo com limitações, as aquisições familiares guardam relação com o padrão de consumo individual ^51^ e, no Brasil, tais dados constituem a única fonte longeva o bastante para permitir a análise de tendência do padrão alimentar da população, uma vez que dados de consumo efetivo de amostra representativa da população só se fazem presentes a partir de 2008–2009 ^52^ . O curto período de referência (uma semana) para a coleta de dados sobre a aquisição de alimentos pelas famílias determina que estimativas da POF devam ser calculadas a partir de agregados de famílias e não de famílias individuais, como feito no presente estudo.

Dentre os pontos fortes deste estudo, destacam-se: o caráter rigorosamente probabilístico da amostra das pesquisas e a representatividade, para as regiões metropolitanas de 1987 a 2017 e nacional a partir de 2003; a distribuição da coleta entre os 12 meses do ano permitindo contemplar variações sazonais do consumo alimentar.

Finalmente, destaca-se que as tendências de evolução do padrão alimentar da população brasileira, com a crescente participação de alimentos ultraprocessados reveladas pelo presente estudo, são consistentes com a participação crescente de doenças crônicas não transmissíveis no perfil de morbimortalidade da população brasileira e, particularmente, com o aumento da prevalência do excesso de peso e da obesidade no país.

## Footnotes

a Instituto Brasileiro de Geografia e Estatística, Diretoria de Pesquisas, Coordenação de Trabalho e Rendimento. Pesquisa de Orçamentos Familiares 2017-2018: avaliação nutricional da disponibilidade domiciliar de alimentos no Brasil. Rio de Janeiro: IBGE; 2020.

b IBGE. SIDRA: banco de dados agregados. Brasília: Instituto Brasileiro de Geografia e Estatística; 2012.

c IBGE. Tabela de composição de alimentos. Rio de Janeiro: Instituto Brasileiro de Geografia e Estatística; 1969.

d Universidade de São Paulo, Food Research Center. TBCA - Tabela Brasileira de Composição de Alimentos. São Paulo: FORC; 2019.
